# TransembleNet: Enhancing vector mosquito species classification through transfer learning-based ensemble model

**DOI:** 10.1371/journal.pone.0322171

**Published:** 2025-05-29

**Authors:** Abdullah Al Maruf, Md. Mahmudul Haque, Rownuk Ara Rumy, Jasmin Jahan Puspo, Zeyar Aung

**Affiliations:** 1 Department of Computer Science and Engineering, Bangladesh University of Business and Technology, Dhaka, Bangladesh; 2 Department of Computer Science and Engineering, Stamford University Bangladesh, Dhaka, Bangladesh; 3 Department of Computer Science and Engineering, Shahjalal University of Science and Technology, Sylhet, Bangladesh; 4 Center for Secure Cyber-Physical Systems, Khalifa University, Abu Dhabi, United Arab Emirates; 5 Department of Computer Science, Khalifa University, Abu Dhabi, United Arab Emirates; Graphic Era Deemed to be University, INDIA

## Abstract

Mosquitoes, which belong to diverse species, play a significant role in ecological systems and public health. The accurate identification (classification) of mosquito species is essential for a comprehensive understanding of their ecological roles, behaviors, and evolutionary patterns. While numerous studies have attempted to classify the mosquito species based on images, the existing works still have limitations. Our research is focused on vector mosquito classification based on deep ensemble transfer learning. Initially, we employed transfer learning via four pre-trained convolutional neural network (CNN) models. Subsequently, we have proposed the TransembleNet (Transfer Learning-based Ensemble Networks) approach, which is a novel method of generating ensemble learning models using four different combinations of three transfer learning models. All the experiments were done using the Nadam and Adam optimizers, and we have also applied data augmentation techniques. Among the four ensemble models, Ensemble Model 2 (composed of InceptionV3, VGG-16, and ResNet-50) performed the best. It exhibits very high precision, recall, F1-score, and accuracy values on the “Mosquito on Human Skin” dataset by Ong and Ahmed and the “Vector Mosquito” dataset by Park *et al*. Our proposed method outperformed the state-of-the-art research works for both datasets.

## Introduction

Diptera, particularly mosquitoes, play a crucial role in transmitting different parasites and pathogens, resulting in hundreds of millions of infections and around 750,000 deaths globally annually [1]. Mosquitoes are regarded as the number one “animal killer” and are among the multiple medically important insect taxa. Recognizing mosquitoes as transmitters of serious diseases like Zika virus, Dengue, malaria, yellow fever, West Nile virus, and Chikungunya is fundamental because these insects pose a serious threat to public health. For example, Anopheles mosquitoes are immediate carriers of malaria, generating 219 million cases worldwide and over 400,000 deaths yearly [2]. Aedes mosquitoes, which transmit Dengue, impact 3.9 billion in 129 countries, with 96 million cases yearly, along with Zika and Chikungunya [3]. Culex mosquitoes are responsible for West Nile, Japanese encephalitis, and lymphatic filariasis. For most diseases spread by mosquitoes, the major preventive measure is still mosquito control because there are no specific vaccinations or medications available [4].

Proper identification of mosquito species is necessary for the effective prevention and management of mosquito-borne diseases. It provides important insights into breeding characteristics and behavioral habits, laying the foundation for targeted prevention techniques [5]. Vector surveillance plays a vital role in evaluating data on mosquito abundance, distribution, and species composition. Despite improvements in vector control, mosquito-borne diseases persist, with aspects such as climate change, insecticide resistance, and the emergence of drug-resistant pathogens responsible for the challenge [6]. According to WHO, harmful mosquito species are commonly found within the genera Aedes, Anopheles, and Culex.

Automatic classification of species is necessary not just for mosquitoes but also for insects, reasonable for various purposes such as environment monitoring, insect diagnostics, forensics, vector epidemiology, etc. [7]. Previous approaches have operated different properties of mosquitoes and insects for automated species classification, including 3D images, sound, genomic data, etc. Traditional methods depending on the morphological characteristics of mosquitoes are time-consuming, labor-intensive, and sensitive to uncertainties deriving from genetic variability. While molecular identification methods are useful, they are costly and require high technical proficiency, making them less accessible for widespread usage. The existing demand for quick and intelligent mosquito species identification exceeds the capabilities of these traditional methods.

Image-based systems have appeared as a promising approach, surrounding three key steps: acquisition of 2D color images, feature extraction, and classification. However, these steps have several challenges. Mosquitoes and insects exhibit broad variations in pose and deformation, in addition to traditional image variations, including scale, rotation, lighting, and cluttered backgrounds. Capturing images of wings in a restrained pose, which is a critical step in the recognition procedure, is time-consuming and laborious. Moreover, the procedure risks damaging wings and further appendages like legs [8].

Identifying mosquitoes is challenging due to their morphological similarities; while some studies identify specific species, most focus on distinct species accessible from laboratory colonies rather than environmental samples. Notably, the current computer vision techniques for mosquito classification have been restrained to closed-set classification scenarios, generally containing at most 17 species [5]. Moreover, considerable genetically detected but formally undescribed species also complicate the landscape.

Recent advancements in Deep Convolutional Neural Networks (DCNNs) have showcased outstanding classification performance in various recognition tasks, spanning document recognition, age and gender classification, galaxy morphology prediction, and vehicle type classification, neurological disease detection, among others [9–11]. DCNN architecture, described by multiple layers of non-linear operations, excels at capturing hierarchical features, addressing the limitations of premature image-based recognition techniques. The confluence of improved computing power, the wave in big data availability, and the growth of machine learning (ML) algorithms have propelled the quick development and application of deep learning techniques in image classification tasks. CNN models, including LeNet-5, AlexNet, GoogLeNet, Visual Geometry Group Network (VGGNet), Residual Network (ResNet), SqueezeNet, etc., have successively been operated for automatic mosquito recognition using images. Consequently, these methods help capture the complicated relationships between pixel points effectively.

However, the existing work on image-based mosquito species classification has some limitations, such as the fact that most authors used limited image samples. There are 3,500 mosquito species, and 90 species are responsible for transmitting diseases [12]. Regardless, most of the previous research worked on a very limited number of classes, and although there are few works done on multiple species, they need to be paid attention to improve. Addressing these challenges is essential for raising the accuracy and dependability of mosquito classification through deep learning methodologies.

### Motivation, objective, and contributions

Mosquitoes cause serious illness, and a large number of people are affected by them. These illnesses are more common in areas with poor hygiene, high population density, and restricted access to medical facilities. To fully comprehend the diverse ecological roles, behaviors, and—most importantly—the potential for mosquito species to transmit disease, one must possess a thorough understanding of these species. Scientific advancement, ecological understanding, and efficient disease control all depend on the classification of mosquitoes. It is essential for identifying specific interventions, keeping an eye on disease vectors, and eventually defending public health against illnesses spread by mosquitoes.

To our knowledge, the CNN models and the pre-trained CNN models have been applied more to this area of image-based mosquito species classification [7,13–17]. However, the use of transfer learning-based ensemble learning has not been explored much. The application of data augmentation is also rare. By increasing the dataset’s diversity, this data augmentation reduces overfitting and strengthens the capacity of models to generalize. The ensemble learning model incorporates the discriminative information from all the constituent base learners, allowing it to make superior predictions. To address the issue of limited data availability, transfer learning models [18,19], also known as pre-trained models, are strong candidates for use as base learners.

Here, the exact problem we are trying to solve is that of automated classification of mosquito species for adult (i.e., post-larvae) mosquitoes by means of computer vision. Our research objective is to develop an accurate and robust classification system using a novel transfer learning-based ensemble learning approach. We limit the scope of our research to using only the still images of different mosquito species in two conditions (landing and smashed) as input data.

Derived by the above objective, the main contributions of this research are as follows.

1. To overcome the problem of small sample sizes in the available dataset, we carefully used data augmentation methods. This method increases the size of the dataset. It adds variability by means of clever transformations, which improves the model’s ability to generalize and extract significant patterns from the augmented samples. Data augmentation plays a vital role in supporting the robustness and performance of the model by creatively addressing the lack of data.
2. We utilized the TransembleNet (Transfer Learning-based Ensemble Networks) approach, which is a novel ensemble learning strategy in our study. Three base learners were used in one ensemble model. We applied four pre-trained CNN models, and when making the ensemble model, we picked three as base learners using a mathematical combination formula. Four distinct ensemble models were produced as a result of this process. To our knowledge, our proposed method is the first to use an ensemble of transfer learning (i.e., pre-trained) models to tackle the problem of mosquito image classification.
3. A thorough assessment of the ensemble models was conducted using a variety of performance metrics and statistical standards. The best ensemble model, which stood out for its exceptional performance, was then evaluated using two publicly available datasets [7,16]. Our proposed ensemble method outperformed the other state-of-the-art methods.
4. Limited attention has been directed towards the simultaneous exploration of smashed conditions in mosquito images and the identification of vector mosquitoes. This study addresses this gap by comprehensively examining smashed mosquito conditions while concurrently classifying vector mosquitoes.

## Background and related work

Detecting mosquito species is an important scholarly concern. Researchers are delving carefully into a number of aspects related to mosquito identification, such as species detection and classification according to developmental stages, such as larvae and eggs.

For the Aedes Larvae Classification and Detection (ALCD) system, the author in [20] employed deep learning (DL) technologies for the pattern of the larva and organized it according to its type. They proposed the CNN model that training the images can result in an accuracy of 73%. However, the accuracy is not sufficient.

In another work, Arista-Jalife *et al*. [21] presented an automated approach for classifying larva images, particularly those of Aedes aegypti and Aedes albopictus. The proposed method achieved 92.85% accuracy. The method not only identifies the region of interest (ROI) in the images but also performs a dual classification, distinguishing between Aedes’ positive and Aedes’ negative instances. To achieve this, the dual algorithm, namely, Single Shot Detector with Deep Neural Network (SSD-DNN), is employed to crop the ROI and categorize the image into a binary outcome with a precision rate of 94.19%. Although the segmentation and detection stages were initially implemented separately, there is potential for process optimization by integrating these stages into a unified framework.

Silvia *et al*. [22] proposed a pre-trained CNN model, namely ResNet-50, that can also identify the mosquito larvae, and the average accuracy is 98.76%. The major drawback of their work is that the proposed model could not identify the larva image if the image is not in the upside body part, and they have used a small dataset containing 160 samples.

Another study [23] worked on mosquito eggs in the pre-stage of the larva. They found similar pixels and used the slice color method for training purposes using learning with CNN to achieve 90% accuracy from mosquito eggs in the pre-stage of the larva. However, they failed in high density of eggs or appeared black elements present in samples.

Detecting mosquito species from images can be critical for different reasons, mainly in the field of public health. Adhane *et al*. [13] used DL and ML methods to classify Aedes Albopictus mosquitoes as Tiger or non-Tiger by using the pre-trained VGG-16 model on the Mosquito alert image dataset, and the proposed model achieved a 94% accuracy. They used an explainable Grad-CAM algorithm to visualize the most discriminant regions of the classified images. The mosquito samples of the dataset are not mentioned in detail.

Another study [24] showed that the dual algorithm works better than DL methods to classify mosquito images by species and sex (male vs. female). Gray Level Co-occurrence Matrix (GLCM) with Random Forest (RF), Support Vector Machine (SVM), and K-Nearest Neighbor (KNN) classifiers were employed. RF with GLCM performed the best and achieved 95.26% accuracy.

Scholars in [15] also presented an Android application to detect the Aedes mosquito species, and their proposed CNN model was able to classify up to 84.87% images accurately. However, the major drawback was that the model only accepts very similar samples as training sets, and accuracy could still be improved by utilizing proper libraries and settings. Also, the model cannot classify multiple classes.

A data augmentation method was used to increase mosquito images [25] and showed a comparison with the CNN model and other ML models. The proposed CNN model achieved 93% accuracy for Aedes, anopheles and Culex mosquito classification. However, the original dataset size was quite small, and the accuracy was poor (70%). Augmentation was essential to achieve a better performance.

Scholars in [14] implemented transfer learning to classify mosquitoes into the corresponding species using Faster R-CNN with InceptionV2 and MobileNet. They reported an average accuracy of 95.19%. The verification needs to be improved since the dataset size is small, and the image quality could be improved. Additionally, the researchers implemented their system with the help of IoT-based devices. But, the major drawback was that they worked on only two classifications of Aedes aegypti (Linnaeus) and Aedes albopictus (Skuse).

In addition to the classification of mosquito species, considerable efforts have been dedicated to the identification of vector mosquitoes, the primary agents responsible for transmitting diseases. Recognizing and categorizing vector mosquitoes is of paramount importance for public health initiatives, as it plays a crucial role in understanding and mitigating the spread of diseases. ML and DL models such as VGG-16 and Extra Tree Classifier (ETC) were applied to identify vector mosquitoes [26]. Two feature selection methods, the ROI-based image filtering and Randomly re-started Incremental Feature Selection (RIFS) methods, have proven helpful in identifying vector mosquitoes. They achieved 99% accuracy. The major drawback of this work is that the system can only detect the presence of two disease classes of mosquitoes, such as the Aedes and Culex.

Scholars in [27] proposed a method to classify Aedes Aegypti using image texture-based feature extraction and SVM, with an accuracy of 80%. Since the system is a manual focus, some improvements can be made, such as extracting better features and adding higher magnification.

Zhao *et al*. [5] introduced a high-definition mosquito dataset with 9,900 images of 17 species and suggested the Swin Transformer-based mosquito species identification model (Swin MSI), which performed with 99.04% accuracy and 99.16% F1-score. Swin MSI was able to reach 100% subspecies-level identification in Culex pipiens Complex and 96.26% accuracy for novel species categorization. However, the authors did not evaluate the model using other performance metrics.

Motta *et al*. [28] carefully selected 823 images collected across the globe from three mosquito species. They applied three pre-trained models: LeNet, AlexNet, and GoogleNet. They observed that GoogleNet performed best, and it obtained 76.2% accuracy.

Goodwin *et al*. [6] constructed a detection algorithm employing 2,696 specimens from 67 species and performing 97.04% accuracy for 16 known species and 89.07% accuracy for novel species. They applied a 3-tier ensemble model involving Xception in Tier 1, RF, SVM, and Wide and Deep Neural Network (WDNN) in Tier 2, and soft voting and Gaussian Mixture Model (GMM) arbiter in Tier 3. However, the study possibly lacks coverage of global species because it employed desiccated mosquito specimens from North America and Africa.

Ong and Ahmed [16] created a dataset of 1,500 mosquito images on the human skin. It consists of three species (A. aegypti, A. albopictus, and C. quinquefasciatus) in two states (landing and smashed). They conducted a pilot test on the dataset to validate the quality of the dataset in terms of the feasibility of deep convolutional neural networks (DCNN) model construction. They utilized a web-based tool, namely Google Teachable Machine 2.0 [29], to train and test a deep learning model with no coding required. A testing accuracy of 92.56% was achieved.

Kumar *et al*. [17] used the dataset created by Ong and Ahmed [16] and applied 6 different DL algorithms, namely, simple DCNN, EfficientNetB7, MobileNetV2, DenseNet121, XceptionNet, and ResNet152V2. They observed that the simple DCNN with hyperparameter tuning provides the best accuracy of 91%.

In Park *et al*. [7], a dataset of 3,600 images of eight mosquito species was created and investigated with the use of DL architectures, such as ResNet-50, VGG-16, and SqueezeNet to classify vector mosquitoes from images, acquiring over 97% accuracy by fine-tuning general features and using data augmentation techniques.

The summary of the related work on image-based mosquito classification is presented in Table 1.

**Table 1:** Existing research work on image-based mosquito classification (A: accuracy; P: precision; F: F1-score).

Ref.	Stage	#classes	Innovations/methods	Results
[20]	Larvae	2	CNN	A: 73%
[21]	Larvae	2	ROI segmentation, SSD-DNN	P: 94.19%
[22]	Larvae	2	ResNet-50 (CNN)	A: 98.76%
[23]	Egg	2	CNN	A: 90%
[24]	Adult	4	GLCM, RF, SVM, KNN	A: 95.26%
[13]	Adult	2	VGG-16 (CNN)	A: 94%
[15]	Adult	2	CNN, Android app	A: 84.87%
[25]	Adult	3	Data augmentation, CNN	A: 93%
[14]	Adult	2	R-CNN, InceptionV2, MobileNet, IoT platform	A: 95.19%
[26]	Adult	2	VGG-16, ETC, ROI, RIFS	A: 99%
[27]	Adult	2	Texture-based features, SVM	A: 90%
[5]	Adult	17	Swin transformer	A: 99.04%, F: 99.16%
[28]	Adult	3	LeNet, AlexNet, GoogleNet	A: 76.21%
[6]	Adult	16	three-tier ensemble, Xception, RF, SVM, WDNN, Soft voting, GMM arbiter	A: 97.04%
[16]	Adult	6	DCNN	A: 92.56%
[17]	Adult	6	DCNN, EfficientNetB7, MobileNetV2, DenseNet121, XceptionNet, ResNet152V2	A: 91%
[7]	Adult	8	ResNet-50, VGG-16, SqueezeNet, Data augmentation	A: 97%

After reviewing the extensive literature, it has become readily apparent that the classification of mosquito species and diseases presents notable research gaps.

1. The datasets’ drawbacks include the preponderance lack of sample sizes in the current datasets, which presents difficulties for training because of data scarcity and imbalance.
2. Many researchers often focused on only a few (2 or 3) classes in their dataset.
3. Majority of the research works had been done using the conventional approach; therefore, the accuracy of the models was quite low.
4. Even while the accuracy of modern models has improved significantly, their effectiveness frequently comes at the expense of time efficiency because of increased computing complexity.
5. Despite few samples in the dataset and the imbalance situation, no proper strategy was employed to solve these issues.
6. The researchers did not do cross-checking on other datasets in order to determine the robustness and generalizability of their model.
7. Previous researchers faced difficulties with model underfitting, overfitting, and biased results, which required further techniques to address these problems.
8. Even though research on diseases and specific mosquito vector species has progressed significantly, there is still a significant gap in treating both at the same time. It is necessary to conduct additional studies in order to help classify species and clarify the complex interactions between the traits of vector species and the dynamics of disease transmission.
9. In this field, ensemble models—especially those that rely on transfer learning—are still comparatively uncommon. Further research is needed to understand how well transfer-learning ensemble combinations perform in identifying vector mosquitoes, and the efficiency and effectiveness of ensemble models need to be focused on.

## Data and methodology

Our research on mosquito species and vector mosquito classification was conducted in multiple stages.

**Fig 1:**
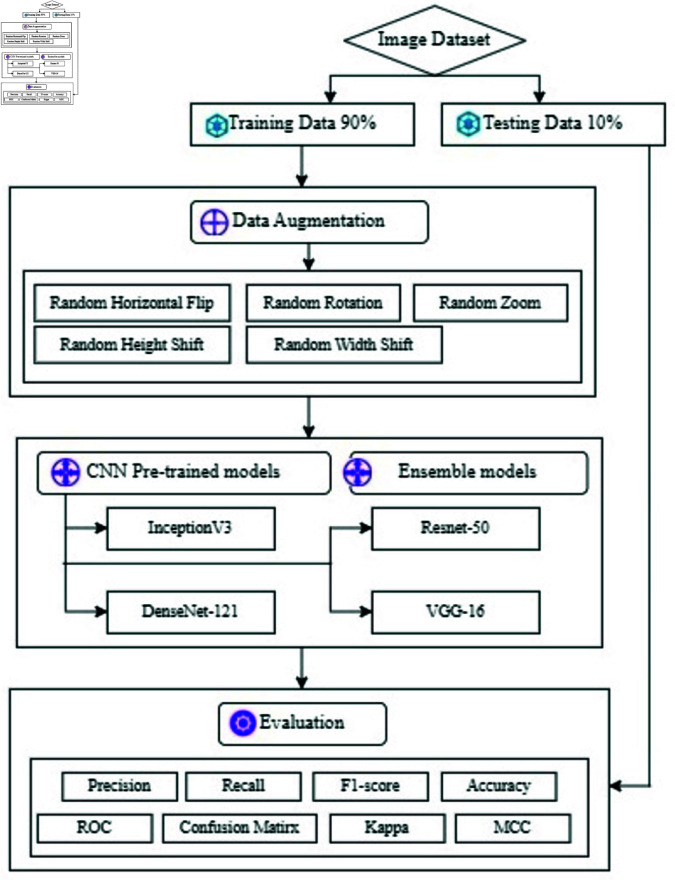
Procedure of the research workflow.

Firstly, we made use of the public dataset that gathered images from different kinds of mosquitoes. Next, in order to increase the number of samples in the dataset, we employed augmentation techniques and image processing techniques to extract pertinent features from the images. With the previously processed data, we then trained pre-trained CNN models. Lastly, to improve overall classification accuracy, we combined the outputs of multiple models using ensemble methods. Next, we used a variety of metrics to assess the models’ performance. Additionally, we evaluated the models’ resilience by putting them to the test on a different validation (a.k.a. testing) dataset and examining how well they performed on data that had never been seen before. The overall procedure of our research is shown graphically in Fig 1.

### Dataset description

The “Mosquito on Human Skin” dataset by Ong and Ahmed [16] is a specialized collection of high-resolution images designed for the development and evaluation of mosquito recognition systems. It is which is available from both Mendeley (https://data.mendeley.com/datasets/zw4p9kj6nt/2) and Kaggle (https://www.kaggle.com/datasets/naiborhujosua/mosquito-on-human-skin), This dataset is a comprehensive and meticulously curated collection of images designed specifically for advancing research in mosquito species recognition on human skin. This dataset is particularly significant due to its focus on three mosquito species: Aedes aegypti, Aedes albopictus, and Culex quinquefasciatus. These species are captured in two distinct conditions. One is their normal landing posture on human skin, and the other one is their appearance when smashed. The dataset contains six classes. It represents a unique combination of species and conditions that comprises a total of 1,500 images, with each class containing 250 images.

**Fig 2:**
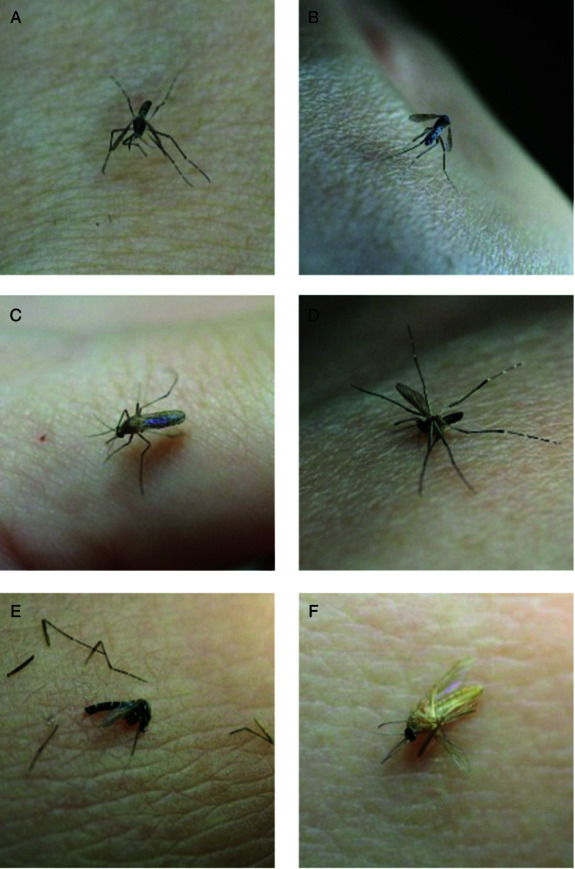
Samples of each class in “Mosquito on Human Skin” dataset (reproduced with permission by the original images’ owners [16]).

Below, we describe the definitions of these three species of mosquitoes in detail, both on “landing” and “smashed”:

- **Aedes aegypti:** The Aedes aegypti mosquito is a significant disease vector known for its black and white stripes shown in Figs 2a and 2d. It originated in Africa and spread worldwide, particularly in tropical and subtropical regions. We have investigated two conditions of it. One is on human skin, and the other is when it’s smashed. This species is a primary transmitter of dengue, Zika, yellow fever, and chikungunya. It prefers laying eggs in water-filled containers close to human habitats, which makes it a prevalent urban health threat. The adult mosquito is small, about 4-7 mm in length. Aedes aegypti’s adaptation to human environments and its breeding in small water collections make it a resilient species. It has developed resistance to many insecticides by complicating control efforts. Control strategies include eliminating breeding sites because they use larvicides and adulticides. It also promotes public awareness of prevention.
- **Aedes albopictus:** The Aedes albopictus is an Asian tiger mosquito that is known for its distinct black and white stripes shown in Figs 2b and 2e. Originally, it was from Southeast Asia. It has spread to many regions worldwide, thriving in both temperate and subtropical climates. This mosquito is also a vector for diseases like dengue, Zika, chikungunya, and West Nile virus. It breeds in various standing water sources, from natural to artificial. Adults are small, about 2 to 10 mm, but they are aggressive biters both day and night. We have used two situations for our dataset. One is the landing position, and another is smashed. Their flight is rapid and erratic, with a high-pitched buzz. Their striped pattern is recognizable even when they are smashed.
- **Culex quinquefasciatus:** The Culex quinquefasciatus is also known as the Southern house mosquito shown in Fig 2c and 2f. It is a common vector for diseases like West Nile virus and filariasis. It is predominant in tropical and subtropical areas and also can adapt well to temperate climates. This species is brown and ranges from 4 to 10 mm in length. Culex quinquefasciatus breeds in stagnant, often polluted water and typically in urban settings. It assumes a hunched posture with its body angled when it lands on human skin. Unlike some other mosquito species, it lacks distinctive markings when it is smashed. Control strategies include eliminating breeding sites and using larvicides and personal protective measures like nets and repellents.

Within the larger fields of entomology and public health, the classification of mosquito species and vector mosquitoes are related. Understanding mosquitoes as both diverse organisms with unique species characteristics and disease carriers explains the relationship between these two classifications. Comprehending the distinct mosquito species that serve as carriers is imperative for focused disease management. Understanding the species of mosquito that carries a disease allows public health initiatives to be concentrated on high-risk regions and populations. So, targeting largely mosquito species classification, we also put our emphasis on on vector mosquito classification.

In this work, we faced a major difficulty brought about by the relative scarcity of data in the dataset. It contains only a total of 1,500 images, which is very limited when compared to general image datasets commonly used in deep learning research, such as CIFAR-100 [30] with tens of thousands of images and ImageNet [31] containing several millions of images. In order to generalize well, deep learning models—especially those for image classification—typically require enormous volumes of data. Nevertheless, our datasets have sample size limitations. This lack of data is a significant challenge because it makes it more difficult for traditional deep-learning techniques to operate at their best. To overcome this difficulty, our work creatively uses both data augmentation methods and ensemble models to incorporate transfer learning techniques. This allows complex characteristics to be extracted from a small dataset.

### Data preprocessing and augmentation

Image preprocessing is a fundamental pipeline phase in our research. Firstly, the image is resized to 299×299×3. Among the datasets, 90% of the data is used for training, and the rest of the samples are used for validation (a.k.a. testing). That is, 1,350 samples are for training, and 150 are for validation. In our research, the dataset is comparatively small. So, we plan to increase the dataset using data augmentation techniques. Data augmentation is a technique used in DL to artificially increase the size and diversity of a dataset by creating new training examples through various transformations or modifications of the existing data. It is commonly used when the available dataset is limited or imbalanced or when the model needs to be more robust to variations in the input data. As the data is limited here, we applied data augmentation techniques in our research. The performance of the learning algorithms is enhanced, and data augmentation techniques avoid overfitting.

The key augmentation strategies we employed through the  tensorflow.keras. preprocessing.image.ImageDataGenerator  class in our research are described below.

- **Random horizontal flip:** This arbitrary augmentation horizontally flips the image. Every image is randomly selected to be left in its original state or flipped horizontally. If flipping is chosen, the image is mirrored by applying the flip function along the horizontal axis.
- **Random rotation:** The image is arbitrarily rotated by a given angle (in radians) via this augmentation. Within the given range, a random angle is selected for every photograph. We employed a maximum of 0.2 radians in our study. The rotate function is then used to rotate the image by this angle.
- **Random zoom:** We use the “Random Zoom” approach, one of the vital augmentation strategies, in this research. For this augmentation, every input image is given a random zoom factor. A zoom factor larger than one creates a reduction or zooming-out effect, offering a broader image perspective. In contrast, a zoom factor less than one generates a magnification or zooming-in effect, effectively focusing on certain portions of the image. This method is applied using the zoom function, which modifies the image based on the factor. The factor is 0.2, and the zoom factor will be randomly selected from a uniform distribution between 0.8 and 1.2.
- **Random height shift:** This process includes moving the pixels within the image up or down to change the image’s height—a vertical displacement effect results from this. Objects in the image can appear to move higher or downwards by adjusting the height. This adds variation to the vertical position of objects, which is particularly helpful in real-world situations where object locations could change. In this case, the function argument 0.2, which denotes the permitted shift’s magnitude, was used. This indicates that the shift factor will be chosen randomly from a uniform distribution ranging from -20% to 20% of the height of the entire image. Assuming that the image’s overall height is represented by the symbol H, the shift factor S will be selected randomly from the interval [-0.2H, 0.2H]. Accordingly, the image’s pixels will be moved up or down by a maximum of 20% of the height of the entire image. The image will be pushed upward or below depending on whether S is positive or negative. A randomly selected shift factor S might be, for instance, -150 pixels if H = 1000 pixels. As a result, the image would have a 150-pixel upward shift.
- **Random width shift:** The difference between this operation and Random Height Shift is that the image’s width is randomly shifted. It adds variation to the items’ horizontal positions in the picture. Additionally, we utilized the number 0.2 in this case, which indicates that a uniform distribution between -20% and 20% of the entire image width will be used to select the shift factor randomly.

**Fig 3:**
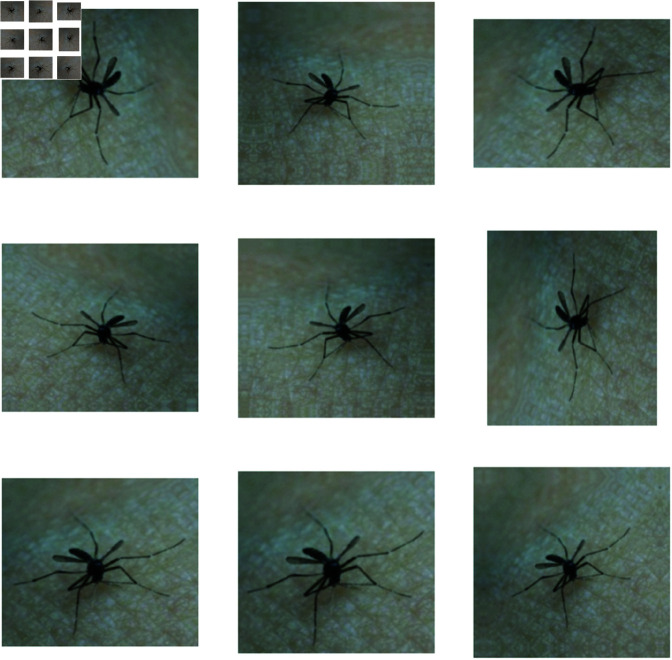
Samples of augmentation. (Augmented images are generated from an original image, which is used with permission by the original image’s owners [16].)

After completing the augmentation, the training samples increased by 95.18% and reached 2,758 samples. A sample of an augmented image is shown in Fig 3.

**Algorithm 1 Data augmentation algorithm for mosquito species classification.**

1: **Input:** Mosquito images

2: **Result:** Augmented dataset with balanced classes

3: **for all** *N* images **do**

4:   ⊳ Mirroring the image horizontally

5:   Apply Random Horizontal Flip with probability 0.5

6:   ⊳ Arbitrarily rotating the image

7:   Apply Random Rotation with maximum angle 0.2 radians

8:   ⊳ Zooming in or out of the image

9:   Apply Random Zoom with factor randomly chosen from [0.8,

1.2]

10:   ⊳ Vertically displacing the image

11:   Apply Random Height Shift with magnitude randomly chosen

from [-20%, 20%]

12:   ⊳ Horizontally displacing the image

13:   Apply Random Width Shift with magnitude randomly chosen

from [-20%, 20%]

14: **end for**

### Pre-trained CNN models (base learners)

In this study, we proposed an unweighted average ensemble model using a combination of pre-trained CNN models as the base learners. We utilized four individual models: (i) InceptionV3 [32,33], (ii) ResNet-50 [34,35], (iii) DenseNet-121 [36,37], and (iv) VGG-16 [38,39]. The reason for selecting those models is their widespread popularity and extensive application in image classification across various domains [40,41]. (As of October 11, 2024, the original papers for Inception [33], ResNet [35], DenseNet [37], and VGG [39] have received 62306, 238992, 47794, and 130804 citations, respectively.)

The pre-trained CNN models were intended for multi-class categorization. It accepts three color channel images with the size IMG_SIZE(299*299) as input. Pre-trained weights from ImageNet are used as a base model in the pre-trained architecture, which is renowned for its efficacy and simplicity. In our work, the pre-trained models’ first fully connected layers were eliminated, and the models were fine-tuned by freezing the layers. Data preprocessing, including data augmentation, is applied to input images. To minimize spatial dimensions, the preprocessed images are sent via a modified pre-trained base and then a global average pooling layer. A dropout layer with a 0.2 dropout rate is added to reduce overfitting. A dense layer with softmax activation is inserted at the end for six classes of classification. The robust pre-trained features are combined with extra layers specifically designed for categorization to create the final model architecture.

The descriptions of the four pre-trained models that we employed in our research are given below.

#### ResNet-50.

ResNet is a principal deep learning architecture, presenting variations with 50, 101, and 152 layers [42], and its learning mechanism revolves around residual representation, where the prior layer’s output operates as the input to the next layer without alterations.

**Fig 4:**
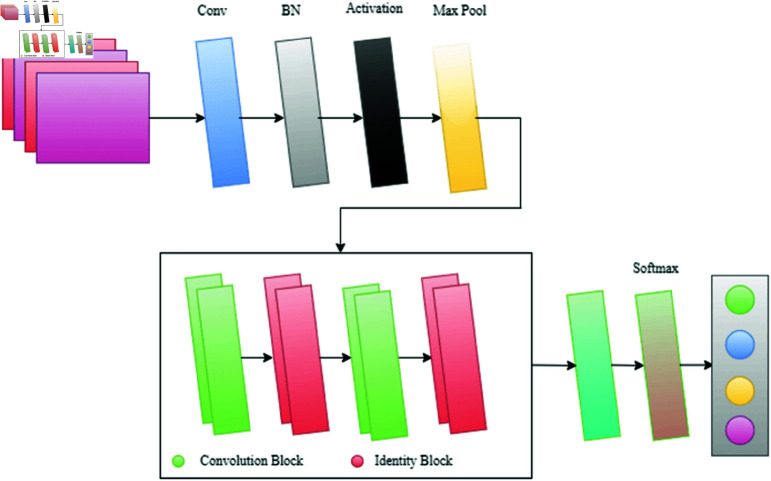
Graphical representation of ResNet architecture.

The architecture includes two key types of blocks: Convolutional and Identity. These blocks, merged, construct the foundation for creating ResNet. Residual entities, categorized as pooling, convolution, and batch normalization layers, form the building blocks, as illustrated in Fig 4.

Mathematically, each unit can be defined using Eqs 1, 2, and 3. Here, *x*_*n*_ is the input, *x*_*n* + 1_ is the output of the *n*-th unit, and *f* is the residual function of the *n*-th unit.

$$y_{n}=h(x_{n})+f(x_{n},W_{n})$$ (1)

$$x_{n+1}=f(y_{n})$$ (2)

$$h(x_{n})=x_{n}$$ (3)

**Fig 5:**
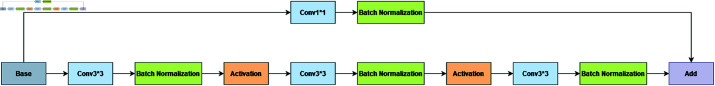
ResNet convolutional block architecture.

**Fig 6:**
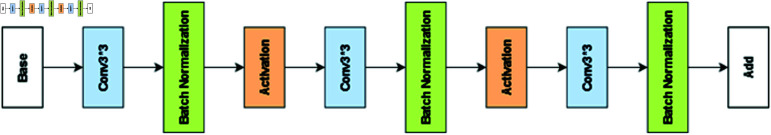
ResNet identity block: A foundational unit in ResNet.

Here, *h*(*x*_*n*_) describes the identity mapping of the activation function. Notably, ResNet uses back-propagation to address the vanishing gradient issue since its residual representation contains short-cut networks. As seen in Figs 5 and 6, these short-cut networks use skip connections, which are similar to basic convolutional layers, to add input values to the output after specific weight layers.

ResNet-50, utilizing 50 parameterized layers with recurrent connections as residual blocks, uses transfer learning. The architecture culminates in a personalized softmax layer for detecting mosquitoes on human skin, enhanced with an L2 regularizer. Noteworthy elements of ResNet-50 include:

- Fully connected layers are previous to the softmax output layer,
- A 7×7 average pooling layer with stride 7,
- 16 residual building blocks,
- A 7×7 convolution layer, and
- A 3×3 max pooling layer with stride 2.

#### DenseNet-121.

DenseNet, an architecture in Convolutional Neural Networks (CNNs), is created to streamline connectivity patterns among layers by allowing direct connections. To overcome the restriction on the maximum information flow, this enables direct connections between each layer and the other layers. In comparison with traditional CNN, there are fewer feature maps in DenseNet than in regular CNN. DenseNet uses feature reuse to maximize the potential of its layers, and it also fixes the problems caused by the gradients. Using a classic feed-forward architecture, the DenseNet associates each layer’s output with the layer before it through a composite operation that combines activation functions, batch normalization, and layer pooling.

The foundational equation governing DenseNet is is given in Eq 4:

$$X_{n}=H_{n}(X_{n-1})$$ (4)

In the ResNet architecture, this equation is extended by containing the skip connection, reformulating as in Eq 5.

$$X_{n}=H_{n}(X_{n-1})+(X_{n+1})$$ (5)

In comparison to ResNet, which expands the equation with skip connections, DenseNet opts for a concatenation approach without the summation of outgoing and incoming feature maps. Therefore, the equation reshapes again into Eq 6.

$$X_{n}=H_{n}([x_{0},x_{1},x_{2},\ldots ,x_{n-1}])$$ (6)

DenseNet employs Dense Blocks as fundamental building blocks within the model structure. These Dense Blocks consist of layers directed as transition layers, which handle down-sampling by including batch normalization. Despite changes in the number of filters, the feature map dimensions stay constant throughout the model. However, the dimensions of each channel experience an expansion as a result of the concatenation of feature maps from every layer.

Every time *H*_*n*_ is employed to produce *k* feature maps in the generalized *n*-th layer as defined in Eq 7.

$$k_{n}=k_{0}+k\cdot (n-1)$$ (7)

The expansion rate of hyperparameter *k* is regulated by the level of information added to the network at each layer. DenseNet demonstrates a distinctive architecture where input volumes and the output of two operations become intricately linked. This connection concerns not only the additive infusion of information into the shared knowledge of the network but also the concatenation of *k* feature maps, thereby constructing an outstanding interweaving of information flow within the network.

#### VGG-16.

The VGG-16 convolutional neural network model is a highly prosperous architecture in computer vision. Well-known for its simplicity and versatility, VGG-16 has become a widely embraced model due to its precise design principles, giving valuable insights for the exploration of deep neural networks.

To maintain spatial resolution post-convolution, the model utilizes 3×3 convolutional filters with both a stride and padding set to 1. Feature extraction concerns the integration of certain convolutional layers with a subsequent 2×2 max-pooling layer, directing with a stride of 2 over a pixel window of 2×2. Individual passage through a pooling layer results in a halving of the input’s spatial resolution.

**Fig 7:**
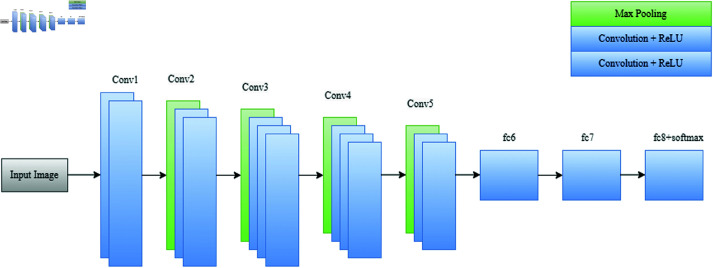
Graphical representation of VGG-16 architecture.

There are three fully connected levels at the end of the architecture. Both of the initial layers have 4,096 channels each, while the number of channels in the third layer varies depending on which particular dataset is being used. The last layer is a softmax layer responsible for classification predictions. ReLU assists in the overall efficacy of the model by serving as the activation function for every layer in the VGG-16 convolutional layer architecture. The deep learning model VGG-16 is constructed of 13 convolution layers, three fully connected layers, and five max-pooling layers, as demonstrated in Fig 7.

#### InceptionV3.

InceptionV3 desires to scale up networks by dealing with the computation as efficiently as possible. It utilizes appropriately factorized convolutions, aggressive regularization, and an advancement in Inception architecture. Inception architecture remains different from conventional approaches to expand network accuracy. The model is carefully created based on four key principles: representational bottlenecks in the network should be ignored; high-dimensional representations are easy to process; low-dimensional embeddings can be utilized for spatial aggregation with no loss in a representative capacity, and the network’s width and depth can be extended concurrently to reach the best performance for a provided amount of computation. Inception architecture stays different from traditional methods of improving network accuracy by following these guidelines.

**Fig 8:**
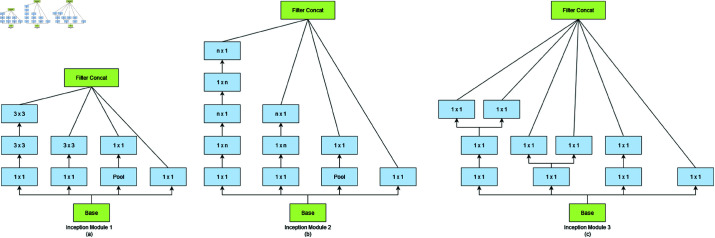
Inception modules: (a) two 3×3 convolutions are employed in place of each 5×5 convolution, (b) factorization of the n×n convolutions, and (c) modules with expanded filter bank outputs.

Table 2 and Fig 8 represent the general structure of the InceptionV3 network architecture.

**Table 2:** General structure of InceptionV3 network architecture.

Layer	Operation	Patch Size	Input Size
1	conv2d(ReLU, BN)	3×3/2	229×229×3
2	conv2d(ReLU, BN)	3×3/1	149×149×32
3	conv2d(ReLU, BN, padded)	3×3/1	147×147×32
4	max pool	3×3/2	147×147×64
5	conv2d(ReLU, BN)	3×3/1	73×73×64
6	conv2d(ReLU, BN)	3×3/2	71×71×80
7	conv2d(ReLU, BN)	3×3/1	35×35×192
8	3 × Inception Module 1	As in Fig 7a	35×35×288
9	5 × Inception Module 2	As in Fig 7b	17×17×768
10	2 × Inception Module 3	As in Fig 7c	8×8×1280
11	max pool	8×8	8×8×2048
12	linear	logits	1×1×2048
13	softmax	classifier	1×1×1000

### TransembleNet: The proposed ensemble model

Ensemble averaging is the process of building multiple models and combining them to produce the desired output, as opposed to creating just one model, in machine learning, especially in deep neural networks. A group of models often outperforms a single model because the individual errors in the models “average out.” Less-than-satisfactory networks are maintained in the ensemble averaging but with reduced weight. Any network can have its bias decreased at the expense of higher variance. It is possible to decrease variance in a group of networks without increasing bias. The concept behind average ensembling is that forecasts are taken from several models and then averaged to arrive at the ultimate forecast.

Deep neural networks with millions to billions of parameters make ensemble learning based on deep learning models more difficult than ensemble learning based on traditional classifiers [43]. Multiple base deep learners require a lot of time and space to train. The huge amount of parameters is thus a problem when using ensemble deep learning methods. When modifying the data level or the baseline model level, ensemble learning strategies are developed. When manipulating data, new training sets are created to train various base learners through sampling or cross-validation data (re-sampling). The ability to reduce the number of parameters used in the ensemble base deep models by choosing the same model and adjusting its parameters sets deep learning apart from traditional or machine learning when it comes to manipulation at the level of base models [44].

Two general types for conducting ensemble learning are (i) Type 1: applying different base models to the same dataset and (ii) Type 2: applying the same base model instances to the same dataset (with different sub-sampling for each instance). We opt to use Type 1 in our research because we cannot afford to sub-sample the dataset, which is already relatively small, and the diversity of base models gives a better generalization of the ensemble model.

The ensemble deep learning system’s strength is determined by its design, which includes choosing the best deep learning models to solve the issue and the right number of base models (three or more). Deep neural networks with millions to billions of parameters make it more difficult for ensemble learning based on deep learning models than ensemble learning based on traditional classifiers [43]. Multiple base deep learners require a lot of time and space to train. Our proposed novel ensemble learning strategy, which is described below, helps solve this problem to some extent.

#### Proposed novel ensemble learning strategy.

**Fig 9:**
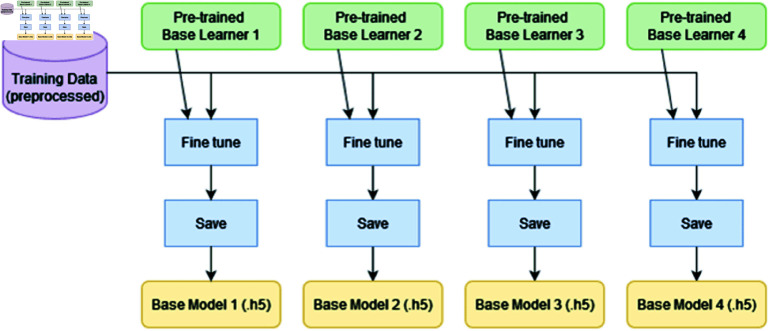
Fine tuning and saving of four base models.

The four pre-trained base learner models are fine-tuned using the training data, and the resultant models are saved (as .h5 files) as shown in Fig 9.

Here, our goal is to create ensembles using those four base models. In order to generate more variety, instead of creating just one ensemble containing all four base models, we opt to create four ensemble models, each containing three different base models (see the subsequent section for more details).

**Fig 10:**
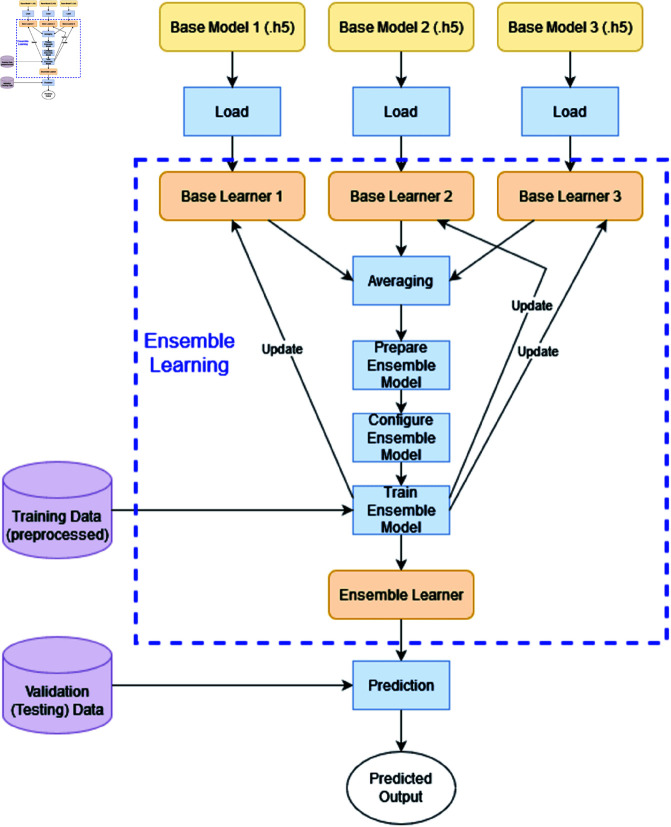
Block diagram of proposed ensemble model.

Let us consider one particular ensemble model (out of four ensembles). Suppose this ensemble contains three base learner models (namely, Base Learner 1, Base Learner 2, and Base Learner 3). Those saved models (.h5 files) from those three models are loaded into memory in order to generate the ensemble model. The input data of shape (299, 299, 3) is defined for a single input layer, which is then transmitted through each loaded model to produce its final outputs. A single composite output is produced by averaging these distinct outputs. The ensemble model’s final classification output is directly derived from the averaged output. By combining the predictions of the three models, this technique makes use of their strengths and may outperform individual models. The ensemble model as a whole (containing the three base models) is trained using the same training data. The weights of the three base models are further updated during the ensemble training process. The block diagram is shown in Fig 10.

Our approach is unique because it builds an ensemble model based on transfer learning specifically designed to handle parameter efficiency and computational complexity. By using pre-trained models with significant amounts of frozen layers, our method dramatically minimizes these complexities compared to existing deep learning models, which frequently exhibit high parameter counts, resulting in significant processing demands during ensemble combinations. We optimize and fine-tune pre-trained models to meet our particular mosquito species classification job by utilizing transfer learning approaches. The development of this customized ensemble model framework based on transfer learning is the main innovation of our work.

This methodology significantly enhances the efficiency and efficacy of ensemble learning in complex classification tasks such as mosquito species identification while also optimizing computational resources. To our knowledge, the use of such an ensemble model idea based on transfer learning for the classification of mosquito species has not been explored before, making it a unique and promising contribution to the field of machine learning in disease vector management and biodiversity conservation.

Our strategy is distinct from other ensemble strategies involving the pre-trained deep learning models such as majority voting [45], average voting [46], weighted voting [47], or combined ensemble outputs being fed into a dense (fully-connected) neural network [48,49]. In all those ensemble models, the base learners (pre-trained models) are frozen and no longer updated once they have been trained with the training data. In our case, another round of end-to-end training of the whole ensemble network was carried out, and the participating base learner models are still updated accordingly.

#### Building ensemble models.

As discussed above, the basic idea of our ensemble learning is an average ensemble model. Our learning procedure involves applying various base models to the same set of data. We have utilized transfer learning as our base model, and for using the different transfer learning models, the structure of the models was different. We have described the base learners’ architecture above. This technique is only possible on deep learning ensemble models where traditional ensemble models cannot perform this learning strategy [43].

In the pursuit of constructing an ensemble model for our research, we sought to explore the various combinations of four pre-trained models. In order to improve generalization and predictive performance, deep learning ensemble methods combine several deep learning models. To improve the performance in deep learning tasks, pre-trained CNN models like VGG-16, InceptionV3, DenseNet-121, and ResNet-50 are assembled by combining their respective strengths. Every pre-trained model excels at capturing various facets of intricate data patterns through their specialized features and hierarchical representations. We developed a more thorough and reliable understanding of the underlying features in the dataset by combining these models. The variety of architectures improves generalization, reduces overfitting, and allows for flexibility with different kinds of input. Through the use of pre-trained models’ transfer learning capabilities, this ensemble approach facilitates efficient knowledge transfer from the massive datasets used in the models’ initial training. We selected three base models at a time to form ensembles with diverse architectures. The calculation of the number of unique combinations was performed using Eq 8.

$$C(n,r)=\frac{n!}{r!(n-r)!}$$ (8)

Here, *n* represents the total number of base models, and *r* denotes the number of models to be selected for each ensemble. For our case, *n* = 4 (the number of available base models) and *r* = 3 (the desired ensemble size). Substituting these values into the formula, we obtained:


$$C(4,3)=\frac{4!}{3!(4-3)!}=4$$

Thus, selecting three models out of the four aforementioned architectures allows for four unique combinations. These combinations represent distinct ensemble configurations with diverse model architectures, contributing to our research’s comprehensive exploration of the model ensemble space.

Here, the ensemble model’s final prediction is obtained by averaging the base learners’ results. Because the DL architectures have low bias and high variance, generalization performance can be improved by simply averaging the ensemble models, which reduces variance among the models. Let *D* represent the pre-trained model set, consisting of {“InceptionV3”, “ResNet-50”, “DenseNet-121”, “VGG-16”}. The images of six different species of mosquitoes from the dataset (*X*_*i*_ and *Y*_*i*_), where *X*_*i*_ is the total number of images and *Y*_*i*_ is the correspondent labels of images *Y* = {“Aedes albopictus landing”, “Aedes albopictus smashed”, “Aedes aegypti landing”, “Aedes aegypti smashed”, “Culex quinquefasciatus landing”, “Culex quinquefasciatus smashed”}.

The size of each image is (299×299). Mini batches are created by dividing the training set by *n*, which lowers the empirical loss and improves the accuracy of the DL models. The base learners’ outputs directly or the classes’ anticipated probabilities using the softmax function are used to average the base learners as shown in Eq 9.

$$P_{ji}=\frac{e^{{\text{softmax}}_{j}(O_{i})}}{\sum_{k=1}^{k}e^{O_{jk}}}$$ (9)

Here, *P*_*ji*_ is the probability outcome of the *i*-th unit on the *j*-th base learner, *O*_*ji*_ is the output of the *i*-th unit of the *j*-th base learner, and *k* is the number of classes. In our case, *k* is 6. As indicated in [35], unweighted averaging makes sense when the base learners’ performance is comparable. In our research, the base learners’ performance is indeed comparable. The changes in the training samples did not lead to significant changes in accuracy in our base learners, which was also a reason to apply unweighted ensemble techniques in our research. This technique is suitable for low model variance. However, because it is influenced by the performance of the overconfident yet weak learners, naive unweighted averaging may lead to sub-optimal performance when the ensemble consists of heterogeneous base learners [50].

**Fig 11:**
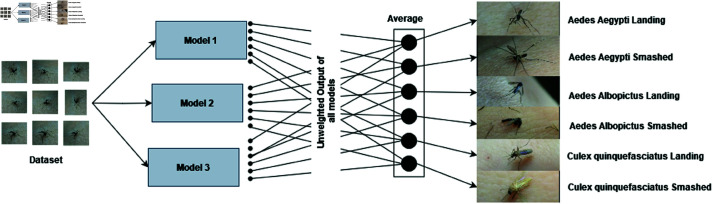
Overview of proposed ensemble model. (The mosquito images are reproduced with permission by the original images’ owners [16]).

The overview of the proposed ensemble model is shown in Fig 11.

**Algorithm 2 Building mosquito species classification ensemble models.**

1: Resize images in dataset (to 299×299)

2: Split dataset into training data and validation data

3: Preprocess training data (using Algorithm 1)

4:

5: ⊳ **Create and fine-tune pre-trained base learner models**

6: *D* = {“InceptionV3”, “ResNet-50”, “DenseNet-121”, “VGG-16” }

7: **for** *i* = 1 to |D| **do**

8:   initialize(*D*[*i*])

9:   fine-tune base learner model *D*[*i*] on preprocessed training data

10:   save model *D*[*i*] (as .h5 file)

11: **end for**

12:

13: ⊳ **Prepare ensemble models through combinations of base learners**

14: ⊳ Calculate number of unique combinations using *C*(*n*,*r*) formula

15: n← number of base learner models (4)

16: r← desired ensemble size (3)

17: num_ensembles←n!/(r!(n−r)!)

18: EM[1]←{D[1],D[2],D[3]}

19: EM[2]←{D[1],D[2],D[4]}

20: EM[3]←{D[2],D[3],D[4]}

21: EM[4]←{D[1],D[3],D[4]}

22:

23: ⊳ **Create, train, and evaluate ensemble models**

24: **for** *i* = 1 to num_ensembles **do**

25:   ⊳ Load saved models

26:   **for** *j* = 1 to *r* **do**

27:    load base model *EM*[*i*][*j*] (from original .h5 file)

28:   **end for**

29:   ⊳ Create ensemble model

30:   initialize input layer: model_input

31:   **for** *j* = 1 to *r* **do**

32:    base_learner_outputs[j]← output of base learner *EM*[*i*][*j*] on

model_input

33:   **end for**

34:   ⊳ Perform unweighted averaging

35:   ensemble_output← average(base_learner_outputs[1…r])

36:   ⊳ Prepare ensemble model

37:   ensemble_model[i]← Model(model_input, ensemble_output)

38:   ⊳ Configure ensemble model

39:   Configure ensemble_model[i] (optimizer: Adam or Nadam, learning

rate: 0.001, loss function: categorical cross-entropy)

40:   ⊳ Train ensemble model

41:   Train ensemble_model[i] on preprocessed training data

42:   ⊳ Evaluate ensemble model

43:   Evaluate ensemble_model[i] on validation data

44:   Print validation accuracy of ensemble_model[i]

45: **end for**

46:

47: ⊳ Select the best ensemble model

48: Identify the ensemble model with the highest validation accuracy

Four ensemble models are created using Algorithm 2 described above. They are:

1. Ensemble Model 1 (EM1): InceptionV3 + ResNet-50 + DenseNet-121
2. Ensemble Model 2 (EM2): InceptionV3 + ResNet-50 + VGG-16
3. Ensemble Model 3 (EM3): ResNet-50 + DenseNet-121 + VGG-16
4. Ensemble Model 4 (EM4): InceptionV3 + DenseNet-121 + VGG-16

## Experimental results

### Experimental setup

The goal of this study’s research strategy is to use an ensemble of pre-trained base learners to categorize mosquito species and identify the vector species based on their images.

First, we split the dataset into 90% for training and 10% for validation (a.k.a. testing). We used 100 epochs to train the ensemble model. We employed comprehensive evaluation criteria to assess the model. Lastly, we applied our model to a validation dataset to test its effectiveness.

We employed the Python programming language for our investigation and Google Colab Pro+ to carry out our experiment. Background execution, a feature of Colab Pro+, allows code to run continuously for up to 24 hours. Idle timeouts only take effect when the code has finished running. The two DL frameworks of TensorFlow and Keras were used to build the model. To manipulate and examine the image data structure, we utilized the Pandas library.

#### Hyperparameters.

The hyperparameters of the proposed method represent the pre-trained models’ estimated computational behavior. In our research, the hyperparameter values were empirically determined. The model was able to achieve an improvement in accuracy at a learning rate of 0.001 when compared to 0.0001, which conversely converged slowly and exhibited poor performance. The optimizer had major implications for performance enhancement. Although Adam and Nadam optimizers exhibited competitive results among themselves, Adam always outperformed Nadam for all metrics. This can be accounted for by Adam’s adaptive learning rate mechanism that smoothly mediates between momentum and adaptive gradient estimation, resulting in faster convergence and better generalization.

The proposed architecture uses Softmax activation functions for the output layer and Rectified linear unit (ReLU) for the hidden layer activation, as stated in Eqs 10 and 11.

$$f(r)=r^{+}=\max(0,r)$$ (10)

$$f(z_{i})=\frac{e^{z_{i}}}{\sum_{i}e^{z_{i}}},\;\forall z_{i}\in z$$ (11)

**Optimizers:** We used two optimizers, namely, (i) Adam [51] and (ii) Nadam [52] optimizers, in building our ensemble models.

1. Adam [51] is a method for first-order gradient-based optimization of stochastic objective functions. The algorithm is based on adaptive estimates of lower-order moments. It is straightforward to implement, is computationally efficient, and has little memory requirements. It is one of the most commonly used optimizers in deep learning [53].
2. Nadam [52] utilizes an advanced momentum algorithm named Nesterov’s accelerated gradient (NAG) instead of the regular momentum used in Adam. The authors presented preliminary evidence suggesting that making this substitution improved the speed of convergence and the quality of the learned models.

**Epochs:** When training a neural network, an epoch is a single run through the whole dataset. Mathematically, one epoch in our dataset with *N* samples (where *N* is the number of our training samples) entails using each of the *N* samples once to update the model’s parameters. Usually, this entails two types of propagation: backward propagation (where gradients are computed and the loss is calculated to update the model’s parameters) and forward propagation (where input data is passed through the network to generate predictions). The model has seen the entire dataset once after finishing one epoch. Several epochs are typically carried out during training to enhance the model’s performance by iteratively updating its parameters using the complete dataset.

**Loss function:** In our multi-class classification vector mosquito and species classification, we make use of the categorical cross-entropy loss function. For each sample *i*, it calculates the difference between the predicted class probabilities ${\hat{y}}_{i}$ and the true class distribution *y*_*i*_. Eq 12 gives the formula for categorical cross-entropy loss.

$$\text{Categorical Cross-Entropy Loss}=-\frac{1}{n}\sum_{i=1}^{n}\sum_{j=1}^{C}y_{ij}\log({\hat{y}}_{ij})$$ (12)

where:

- *n* denotes the number of samples,
- *C* denotes the number of classes,
- *y*_*ij*_ is the indicator function that equals 1 in the event that sample *i* truly belongs to class *j* and 0 otherwise,
- ${\hat{y}}_{ij}$ represents the expected probability that sample *i* belongs to class *j*.

The experimental hyperparameters details are shown in Table 3.

**Table 3:** Hyperparameters of ensemble models.

Image size	299*299
Epochs	100
Learning rate	0.001
Optimizer	Adam, Nadam
Loss function	Categorical crossentropy
Class weight	Class weights based on class sizes
Use multiprocessing	True
Base architecture	Pre-trained models
Batch size	128
Model type	Multiclass classification
Hidden activation	Relu
Output activation	Softmax
Random State	1

#### Trainable and non-trainable parameters.

Trainable and non-trainable parameters make up the parameter spaces for our pre-trained models (base models of the ensemble). The number of parameters (a.k.a. weights) that the backpropagation algorithm can modify is indicated by the term “trainable parameters.” The non-trainable parameters, on the other hand, are static values that are not changed during training. The numbers of parameters in our base pre-trained CNN models are displayed in Table 4.

**Table 4:** Total parameters, trainable parameters, and non-trainable parameters of the pre-trained CNN models.

Model	Total params	Trainable params	Non-trainable params
InceptionV3	21,817,127	1,949,703	19,867,424
ResNet-50	23,602,055	14,343	23,587,712
DenseNet-121	7,044,679	3,058,759	3,985,920
VGG-16	14,718,279	3,591	14,714,688

#### Evaluation metrics.

In order to thoroughly evaluate the effectiveness of our model, we utilized various statistical and graphical techniques.

**Fig 12:**
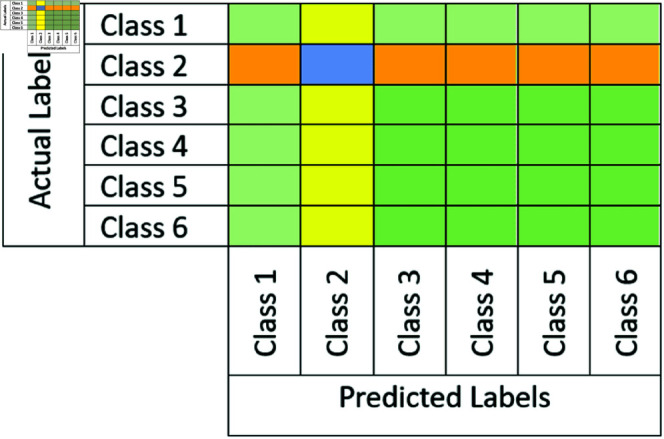
An example of confusion matrix with 6 classes. If the current class *i* is 2, blue represents *TP*, green *TN*, yellow *FP*, and orange *FN*.

Firstly, we plot a confusion matrix (explained in Fig 12) for each classification exercise. We evaluated our pre-trained models and the ensemble models using the evaluation metrics of accuracy, precision, recall, and F1-score, which are all derivable from the confusion matrix. Moreover, we assessed our ensemble model using additional criteria such as Cohen’s Kappa (κ) indicator, Matthews correlation coefficient (MCC), receiver operating characteristic (ROC) curve and its area under the curve (AUC), and precision-recall curve.

This multifaceted approach to evaluation allowed us to gain a more comprehensive understanding of our model’s strengths and weaknesses. By analyzing both statistical and graphical data, we identified areas in which our model excelled and areas in which further improvement was necessary. Furthermore, our use of additional evaluation criteria, such as Kappa, MCC, and AUC, allowed us to assess our model’s performance in a more nuanced and sophisticated manner. This, in turn, allowed us to draw more meaningful conclusions about our model’s effectiveness in real-world scenarios.

The precision of positive predictions is measured. It is the proportion of accurate positive predictions to all of the model’s positive predictions. Recall gauges the capacity to record every positive instance; it is also referred to as sensitivity or true positive rate. It is the proportion of all actual positive occurrences to all true positive predictions. The harmonic mean of recall and precision is known as the F1-score; the other name for it is the F-score or F-measure. The model’s overall correctness is measured by accuracy. It is the proportion of cases that were accurately predicted for all instances. They are calculated as follows:


$$\text{Precision}=\frac{TP}{TP+FP},\;\text{Recall}=\frac{TP}{TP+FN}$$

$$\text{F1-score}=\frac{2\times (\text{Precision}\times \text{Recall})}{\text{Precision}+\text{Recall}},\;\text{Accuracy}=\frac{TP+TN}{TP+TN+FP+FN}$$

When considering class *i* (1≤i≤C, where *C* = 6 is the number of classes, we regard *i* as the positive class and the remaining classes as a negative class.

Here,

- *TP* = no. of true positives (no. of instances belonging to class *i* correctly predicted as class *i*),
- *FP* = no. of false positives (no. of instances belonging to other classes incorrectly predicted as class *i*),
- *TN* = no. of true negatives (no. of instances belonging to other classes correctly predicted as other classes), and
- *FN* = no. of false negatives (no. of instances belonging to class *i* incorrectly predicted as other classes).

The concepts of *TP*, *FP*, *TN*, and *FN* are illustrated in Fig 12.

For a multi-class classification task with *C* number of classes (*C* = 6 in our case), *C* sets of metrics for precision, recall, F1-score, and accuracy are generated, and the average metric values are taken.

For classification tasks, Cohen’s Kappa is a statistical indicator of inter-rater agreement. It takes into consideration the chance that an agreement could occur. The value of Kappa is a number between -1 and 1, where -1 denotes perfect disagreement, 0 represents an agreement that is equal to chance, and 1 indicates perfect agreement.

To handle multiple classes, Cohen’s Kappa for multi-class classification can be modified from the binary case. When dealing with multiple classes, the Kappa statistic is usually calculated by taking into account the degree of agreement and disagreement that exceeds chance in each class. The multi-class Cohen’s Kappa formula is as follows:


$$\kappa =\frac{P_{o}-P_{e}}{1-P_{e}}$$

where:


$$P_{o}=\frac{\sum_{i=1}^{C}{\text{conf}}_{ii}}{\text{total instances}}$$

and


$$P_{e}=\frac{\sum_{i=1}^{C}({\text{marginal row}}_{i}\times {\text{marginal column}}_{i})}{(\text{total instances}{)}^{2}}$$

where:

- ${\text{conf}}_{ii}$ is the number of instances where the predicted class and the true class are both *i*.
- ${\text{marginal cow}}_{i}$ is the sum of row *i* in the confusion matrix.
- ${\text{marginal column}}_{i}$ is the sum of column *i* in the confusion matrix.
- total instances is the total number of instances.

To handle multiple classes, MCC for multi-class classification can be modified from the binary case. Usually, in a multi-class scenario, the confusion matrix for each class is taken into account when computing the MCC. The multi-class MCC formula is as follows:


$$\text{MCC}=\frac{\sum_{i=1}^{C}(TP_{i}+FP_{i})\times (TP_{i}+FN_{i})\times (TN_{i}+FP_{i})\times (TN_{i}+FN_{i})}{\sqrt{\sum_{i=1}^{C}TP_{i}\times TN_{i}-FP_{i}\times FN_{i}}}$$

where:

- *C* is the number of classes.
- *TP*_*i*_: True positives for class *i*.
- *TN*_*i*_: True negatives for class *i*.
- *FP*_*i*_: False positives for class *i*.
- *FN*_*i*_: False negatives for class *i*.

### Generalization results of base models

We have utilized four different pre-trained models named InceptionV3, ResNet-50, DenseNet-121, and VGG-16 to classify images into six different classes related to the three mosquito species in two states (landing or smashed).

To investigate the generalization capabilities of those four base models, we have presented the training and validation accuracies (after 100 epochs) of these models in Table 5.

**Table 5:** Accuracy of pre-trained CNN models.

Model	Training accuracy	Validation accuracy
InceptionV3	90.30%	84.00%
ResNet-50	78.37%	85.33%
DenseNet-121	91.48%	88.00%
VGG-16	45.85%	56.67%

**Fig 13:**
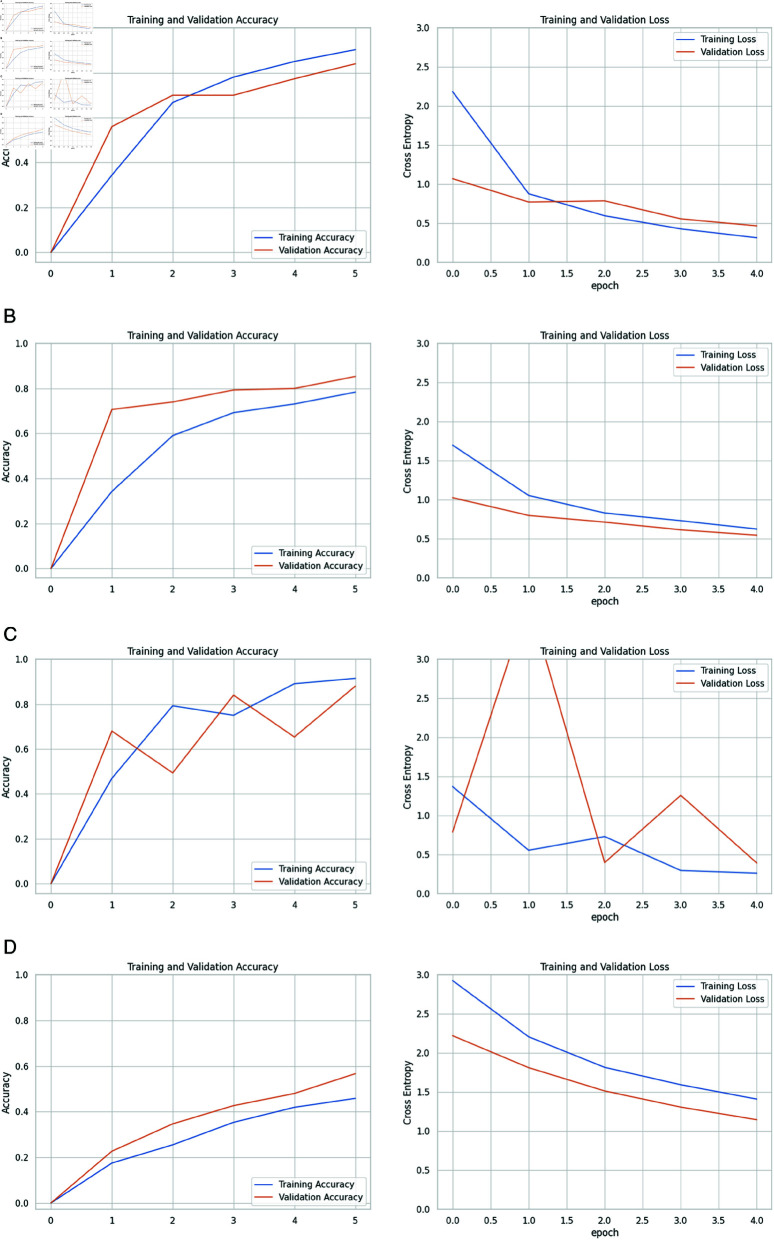
Training/validation loss and Training/validation accuracy of pre-trained Models: (a) InceptionV3, (b) ResNet-50, (c) DenseNet-121, and (d) VGG-16.

Fig 13 displays the outcome of “Training and validation accuracy” and “Training and validation loss” of the four pre-trained CNN models in every epoch.

#### InceptionV3.

The pre-trained model InceptionV3 consists of 48 layers, including convolutional layers, pooling layers, fully connected layers, and auxiliary layers. It has achieved 90.30% training accuracy and 84% validation accuracy, with the Nadam optimizer having 21,817,127 Total parameters, 1,949,793 trainable parameters, and 19,867,424 non-trainable parameters. The difference between training and validation accuracy is 6.3%, which indicates the model might be overfitted.

Fig 13a has described the InceptionV3 five-epoch training process to represent a full cycle through the dataset. In the first epoch, the model achieved a validation accuracy of 56.00% and 1.0716 validation loss with a training accuracy of 34.37% and a training loss of 2.1857. Then, both accuracies improved significantly in the second epoch, with the validation accuracy reaching 70.00% and the training accuracy at 66.74%. The loss reached 0.8787 for training and 0.7000 for validation loss. While the training accuracy increased to 78.07% and loss decreased to 0.5971 in the third epoch, the validation accuracy remained at 70.00%, though the validation loss has been slightly increased to 0.7875. Then, after the fourth epoch, the validation accuracy improved to 77.33%, and the training accuracy rose to 85.04%, with the loss reducing further to 0.4291 and 0.5574 for both training and validation losses. In the first two epochs, the model exhibited underfitting, suggesting that it hasn’t yet learned the complexities of the data. On the other hand, overfitting began to emerge in the third epoch because the training accuracy surpassed the validation accuracy. Finally, the model achieved its best performance in the fifth epoch with a training accuracy of 90.30%, a validation accuracy of 84.00%, and the lowest training loss of 0.3156, where the validation loss is 0.4673. The model was saved after each epoch that saw an improvement in validation accuracy. The training process was computationally intensive, with each epoch taking between 405 to 449 seconds to complete.

#### ResNet-50.

ResNet-50 has a total of 23,602,055 parameters, 1,443 trainable parameters and 23,587,712 non-trainable parameters with a depth of 50 layers. The training accuracy obtained by ResNet-50 is 78.37%, and the validation accuracy is 85.33%. In this case, an underfitting issue was presented.

Fig 13b shows RenseNet-50 performance in every epoch. In epoch 1, the model leaped from an undefined initial validation accuracy of 70.67% with 1.0227 validation loss. This significant upturn is coupled with a modest training accuracy of 34.22% and a loss of 1.6998. This epoch indicates underfitting as the model has a low training accuracy compared to a higher validation accuracy. Epoch 2 climbed to 74.00% in validation accuracy with training accuracy, which has raised to 59.04%, and losses have been reduced to 1.0560 validation loss and 0.8004 training loss. The model hit a stride by reaching a validation accuracy of 79.33% and training accuracy of 69.26%, where losses are 0.8316 for training and 0.7152 for validation loss. The model’s consistency and improvement are evident in Epoch 4. It achieved 80.00% in validation accuracy where the loss is 0.6164 and a training accuracy of 73.11% with a loss of 0.7326. Lastly, epoch 5 culminated this upward trajectory, marking the peak with an impressive 85.33% validation accuracy and a solid 78.37% training accuracy, where the loss is 0.6261 for training and 0.5476 for validation.

#### DenseNet-121.

DenseNet-121 has performed very well by getting 91.48% training accuracy and 88% validation accuracy with the help of the Nadam optimizer. The validation accuracy is reasonably close to the training accuracy, and it indicates effective generalization. DenseNet-121 has comprised 3,985,920 non-trainable parameters, 3,058,759 trainable parameters, and 7,044,679 total parameters, having a total of 121 layers where each layer is connected to every other layer in a dense block to contribute to its depth and efficiency in feature learning.

Throughout five intriguing epochs, DenseNet-121 (Fig 13c) validation accuracy has soared to 68% with a promising debut complemented by a 46.89% training accuracy where the losses are 1.3716 for training and 0.7898 for validation. In the last and final epoch, the model reached its zenith with a stellar 88% validation accuracy and 91.48% training accuracy, where the training loss is 0.2637 and the validation loss is 0.3938. The model’s validation accuracy is 88%.

#### VGG-16.

VGG-16 presented the lowest performance among all the pre-training models, with the Nadam optimizer containing 14,718,279 total parameters, where 3,591 are trainable and 14,714,688 are non-trainable. The model has a total of 16 layers, including 13 convolutional layers and 3 fully connected layers. But, when we customized the model, we used one fully connected layer in our research. The accuracy of both training and validation accuracy values is much lower than other pre-trained models. It has yielded 45.85% training accuracy and 56.67% validation accuracy. The model has appeared to be underfitted as validation accuracy is relatively lower than training accuracy, which indicates that the model is not capturing the underlying patterns in the data effectively.

We have presented the five-epoch journey of VGG-16 in Fig 13d. Starting with Epoch 1, the model showed a validation accuracy of about 22.67% and a training accuracy of 17.48%, where training loss is 2.9269 and validation loss is 2.2223. There’s a consistent improvement as the epochs progress. The validation accuracy rises to 34.67%, with the training accuracy at 25.48% in epoch two, having a 2.2080 training loss and 1.8132 validation loss. By moving to epoch 3, the model reached 42.67% in validation accuracy and 35.33% in training accuracy. Here, 1.8170 is the training loss, and 1.5151 is the validation loss. Having a training loss of 1.5944 and validation loss of 1.3085 in the fourth epoch, the validation accuracy goes up to 48.00% and the training accuracy to 41.93%. In final epoch 5, the model hits a validation accuracy of 56.67% and a training accuracy of 45.85% with a validation loss of 1.1472 and a training loss of 1.4109. Though the model does improve its accuracy over time, the training accuracy remains relatively low compared to the validation accuracy, suggesting that the model’s capacity may be too limited to grasp the nuances of the training dataset fully. So, despite the steady increase in accuracy throughout the five epochs of training the VGG-16 model, persistent underfitting was evident.

### Testing results of base models

**Fig 14:**
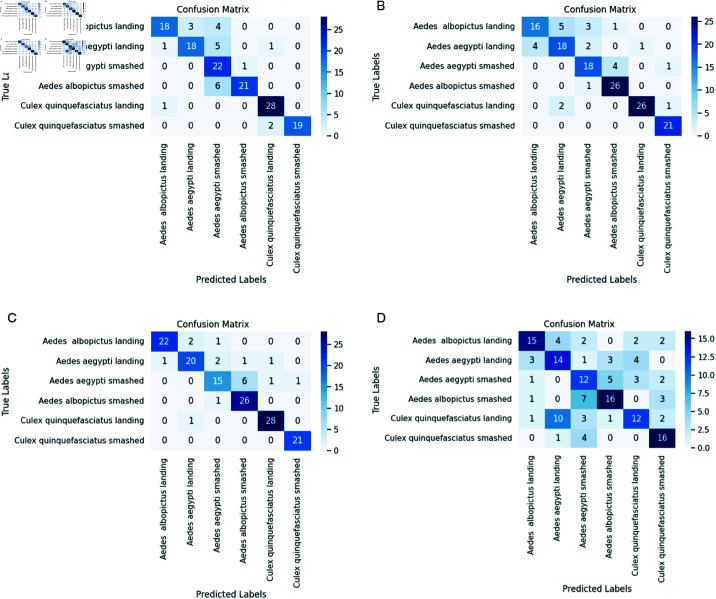
Confusion matrices of pre-trained CNN Models: (a) InceptionV3, (b) ResNet-50, (c) DenseNet-121, and (d) VGG-16 (contd.).

In addition to the testing accuracy, we investigate the four pre-trained CNN models’ other performance evaluation metrics, namely precision, recall, and F1-score. In order to do that, first, we generate the confusion matrices of those four models on the testing (validation) data as displayed in Fig 14.

From the confusion matrices, the performance evaluation metrics (Precision, recall, and F1-score) are calculated. The average values for each evaluation metric for all the classes are presented in Table 6.

**Table 6:** Precision, recall, F1-score of the pre-trained CNN models.

Model	Precision	Recall	F1-score
InceptionV3	87.00%	84.00%	84.00%
ResNet-50	86.00%	85.00%	85.00%
DenseNet-121	88.00%	88.00%	88.00%
VGG-16	58.00%	58.00%	57.00%

#### InceptionV3.

Firstly, InceptionV3 achieved a precision of 87.00%, meaning that 87% of the time, it is correct in predicting a class. With a recall of 84.00%, the model is able to capture 84% of the relevant instances. The F1-score, which harmonizes precision and recall, also stands at 84.00%, suggesting a balanced performance.

#### ResNet-50.

Another pre-trained model, ResNet-50, performs slightly lower in terms of precision compared to InceptionV3. The model has achieved 86% precision. However, the recall value of the ResNet-50 model is higher, almost 1%, than that of InceptionV3. This model’s performance is more balanced than that of InceptionV3. The harmonic mean of this model is 85%, which is 1% higher than the previous one.

#### DenseNet-121.

DenseNet-121 achieved the same value for both precision and recall, about 88%, which is higher than the previous models. Also, this model is able to obtain a perfectly balanced F1-score, which is 88%. That typically means that the pre-trained model DenseNet-121 performed very well, especially in terms of both identifying true positives and avoiding false positives.

#### VGG-16.

At last, we applied VGG-16 as a pre-train CNN model. But in this case, we got much lower values. The VGG-16 performs noticeably worse in terms of precision, recall, and F1-score. VGG-16 got only 58% precision and recall value, and the F1-score is 57%, whereas InceptionV3, ResNet-50, and DenseNet-121 achieved higher scores. VGG-16 obtained 18-20% lower precision and 16-20% lower recall than the other three pre-trained models.

So, our experiments showed that DenseNet-121 is the best pre-trained CNN model among those that have been applied; it offers the highest F1-score, recall, and precision. Conversely, VGG-16 performs the worst, having the lowest F1-score, recall, and precision. These findings highlight that to classify the mosquito species, we need extensive experiments to achieve better performance.

### Generalization results of ensemble models

From the previous analysis, we have found that DenseNet-121 gives the best results among the four pre-trained models. Also, InceptionV3 and DenseNet-121 showed overfitting, and VGG-16 and ResNet-50 models showed underfitting. To overcome the situation of a pre-trained model and achieve better accuracy, we proposed ensemble models (EM1, EM2, EM3, and EM4). The models are a combination of previously trained base models.

The training and validation accuracies of the ensemble models, representing their generalization capabilities, are shown in Table 7.

**Table 7:** Accuracy of four ensemble models with different optimizers.

Ensemble model	Nadam optimizer		Adam optimizer	
	Training accuracy	Validation accuracy	Training accuracy	Validation accuracy
EM1	99%	93%	100%	95.33%
EM2	99%	96%	99.56%	96%
EM3	99%	92%	99.41%	92%
EM4	97%	91%	99.85%	94%

**Fig 15:**
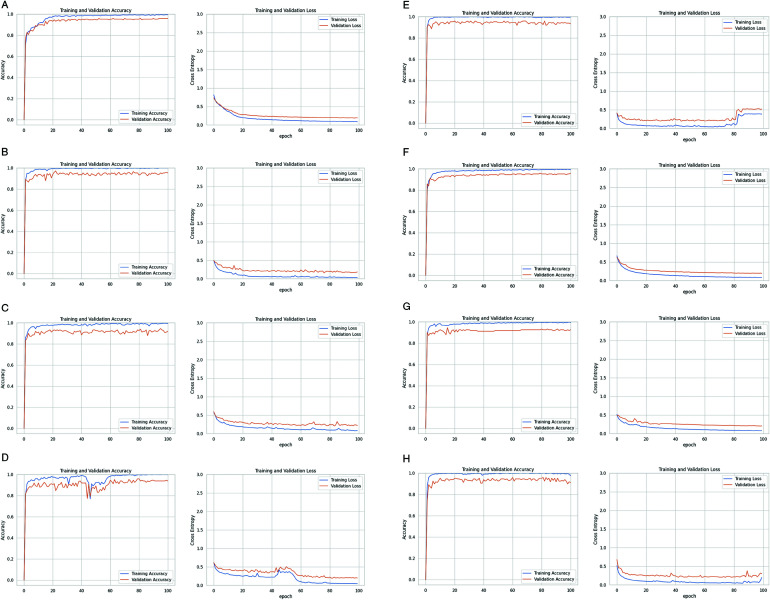
Training/validation loss and training/validation accuracy of four ensemble models using Adam and Nadam optimizers.

The graphical representations of training, validation accuracies, and losses for different epochs are shown in Fig 15.

#### Ensemble Model 1 (EM1).

The first model, EM1, is the combination of InceptionV3, ResNet-50, and DenseNet-121, showing about 99% and 93% accuracy on training and validation, respectively, using the Nadam optimizer. EM1’s training and validation accuracy increased significantly using the Adam optimizer. We achieved 95.33% validation accuracy when Adam is used as an optimizer in this model. However, EM1 still outperformed the best-performing single pre-trained model DenseNet-121.

#### Ensemble Model 2 (EM2).

The second one, EM2, which is a combination of (VGG-16 + ResNet-50 + InceptionV3), gives the same training accuracy as the first one but reduces 3% overfitting. The validation accuracy is 96% by the Nadam optimizer. Using the Adam optimizer, the model achieved the same validation accuracy, whereas training accuracy slightly increased.

#### Ensemble Model 3 (EM3).

The third ensemble model, EM3, performs worse than EM1 and EM2. This model is a combination of the ResNet-50, VGG-16, and DenseNet-121 weak learners. However, the model’s accuracy is 99% and 92% in training and validation accuracy for both optimizers.

#### Ensemble Model 4 (EM4).

Finally, the fourth ensemble model, EM4, which is the combination of InceptionV3, VGG-16, and DenseNet-121, performs best when the optimizer is used as Adam. The model’s validation accuracy is 91% for Nadam and 94% for Adam. The analysis shows that the best-performing pre-trained model (DensNet-121) combinations give lower accuracy in the ensemble model most of the time.

Among the four ensemble models, EM2 performs best and does not suffer from any overfitting issues. Notably, DenseNet-121 was not involved in the combination of EM2. The worse ensemble model performances were observed in the combinations that involved the DenseNet-121 model. Using the Adam as an optimizer performed best in our research. However, all of the ensemble models outperformed all the pre-trained CNN models. So, the experiments showed that the combination of weak learners increases the performance in our research.

### Testing results of ensemble models

**Fig 16:**
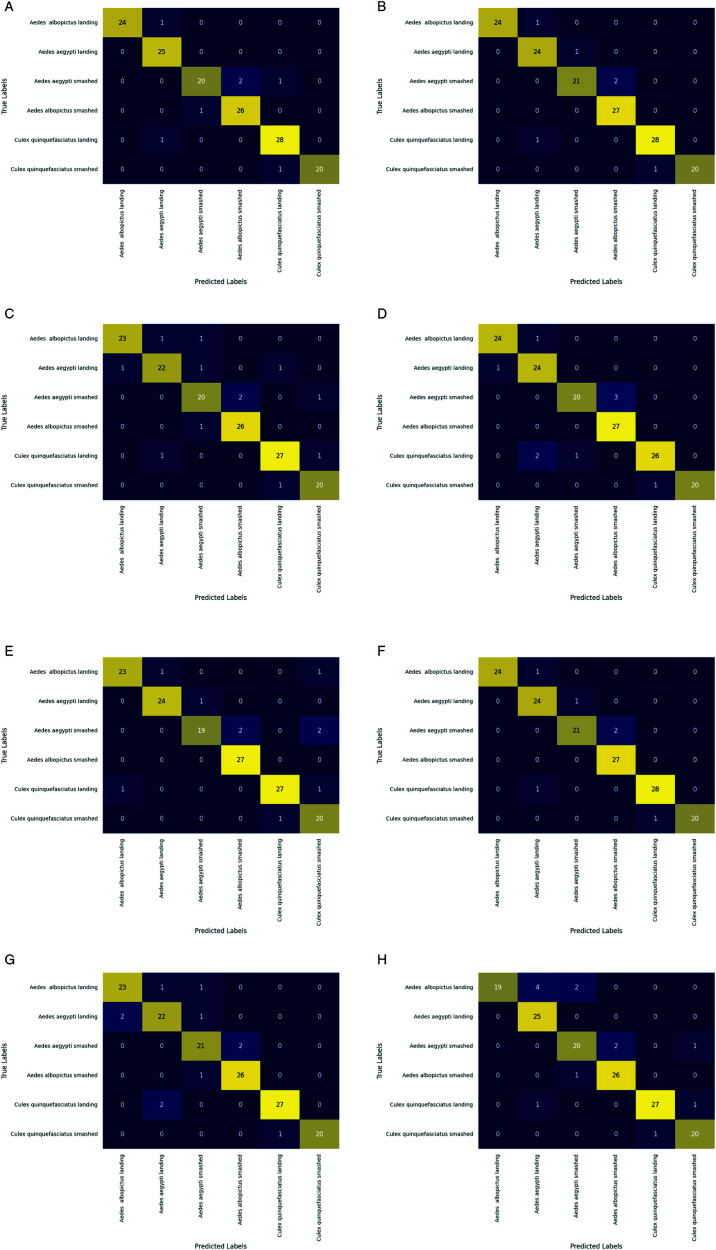
Confusion matrices of four ensemble models using Adam and Nadam optimizers.

The confusion matrices of the four ensemble models on the testing (validation) data are shown in Fig 16.

The precision, recall, and F1-score of the ensemble models are shown in Table 8. To gain a more nuanced understanding of the performance of the four models, it is imperative to analyze their details thoroughly.

**Table 8:** Precision, recall, and F1-score of four ensemble models.

Ensemble model	Nadam optimizer			Adam optimizer		
	Precision	Recall	F1-score	Precision	Recall	F1-score
EM1	93%	93%	93%	96%	95%	95%
EM2	96%	96%	96%	96%	96%	96%
EM3	93%	93%	93%	92%	92%	92%
EM4	92%	91%	91%	94%	94%	94%

#### Ensemble Model 1 (EM1).

EM1 achieved 96% precision, 95% recall, and 95% F1-score with Adam optimizer. The Nadam optimizer, a variant of the Adam optimizer, produced 93% precision, recall, and F1-score, which is 3% lower than the Adam optimizer performance.

#### Ensemble Model 2 (EM2).

EM2 demonstrated the robustness of the Adam optimizer in optimizing the model’s parameters and striking a harmonious balance between precision and recall, with an astounding 96% precision, recall, and F1-score. In terms of precision, recall, and F1-score, the model performed better than EM1, but more significantly, it maintained a balanced performance.

#### Ensemble Model 3 (EM3).

EM3, using the Nadam optimizer, consistently produced results with 93% precision, recall, and F1-score. Still, the model’s performance was inferior to that of EM1 and EM2.

#### Ensemble Model 4 (EM4).

EM4 has marginally better performance than EM3 using the Adam optimizer as well. EM4 performed worse than EM1 and EM2 with 94% precision, 94% recall, and 94% F1-score.

Out of the four models, EM2 with the Adam optimizer is the most efficient and well-balanced model, with the best precision, recall, and F1-score. Some of the classification results produced by EM2 are demonstrated as examples in Fig 19.

**Fig 17:**
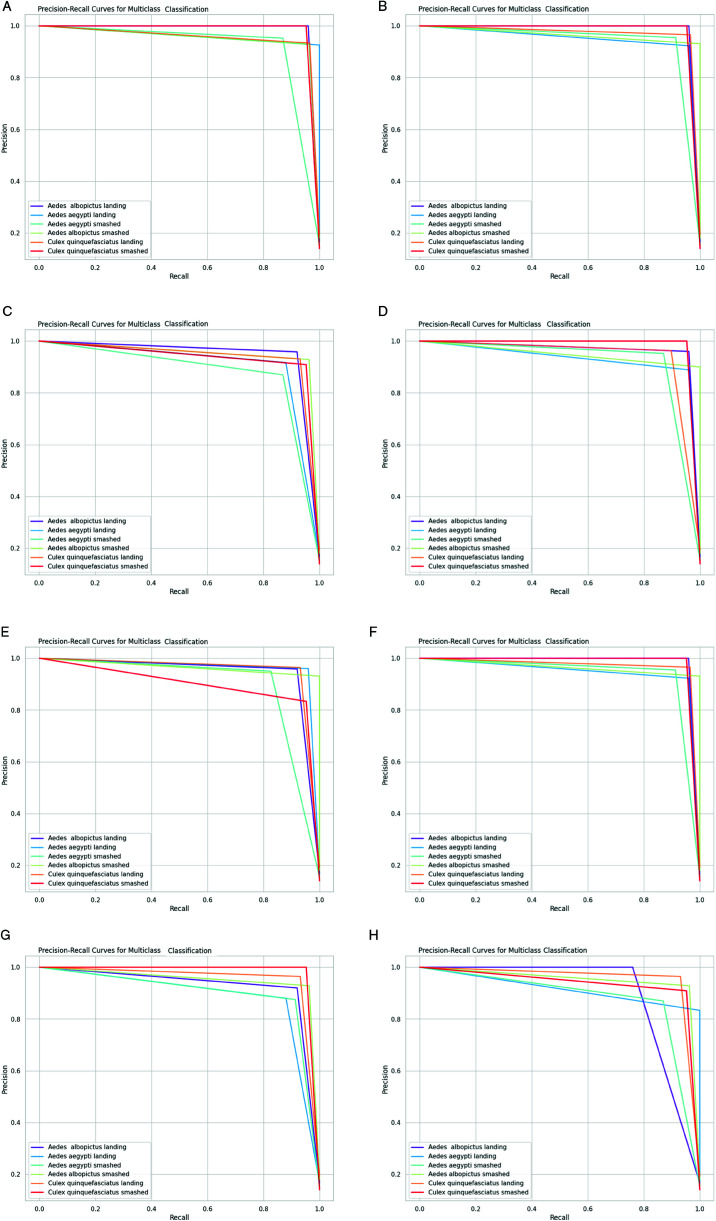
Precision-recall curves of four ensemble models using Adam and Nadam optimizers.

**Fig 18:**
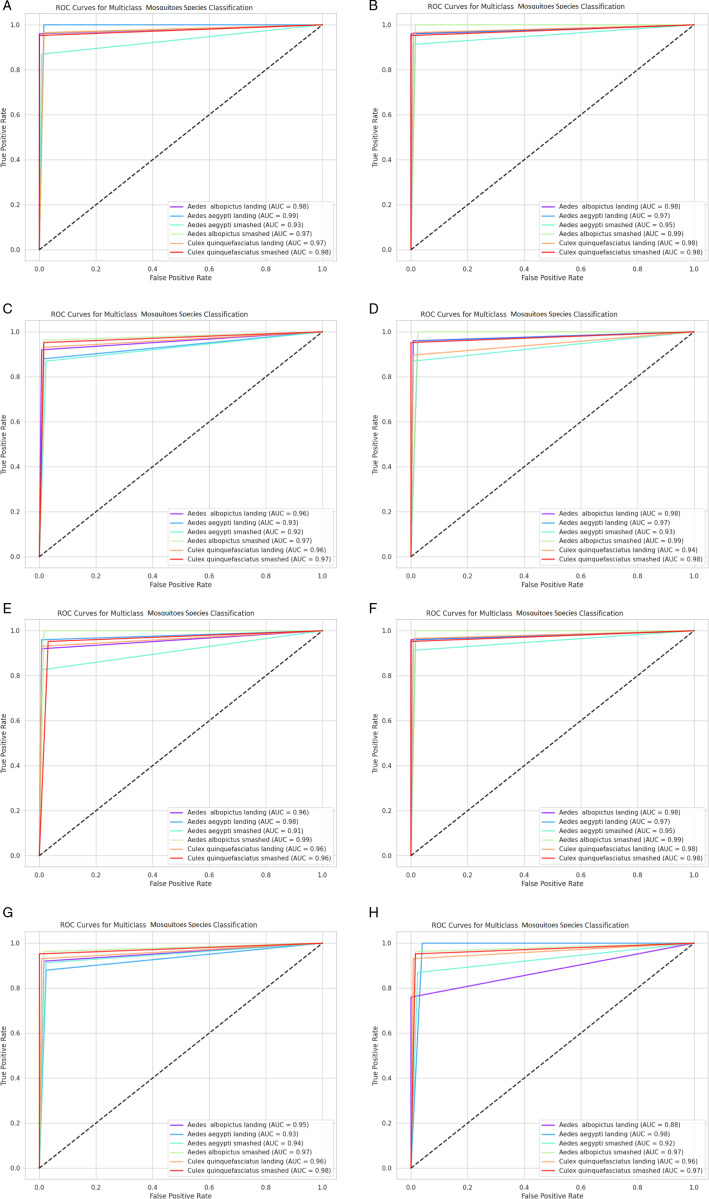
ROC curves of four ensemble models using Adam and Nadam optimizers.

EM1 exhibited competitive results with EM2, using the Adam optimizer. EM3 and EM4 perform marginally worse than the other two, with EM3 being the least optimal, especially when it comes to recall and F1-score.

#### Kappa and MCC results.

To evaluate the model extensively, We have also calculated the Kappa (κ) and MCC values of our model, which are shown in Table 9. The findings provided insight into the models’ consistency and dependability in making predictions.

**Table 9:** Kappa and MCC values of four ensemble models.

Ensemble Model	Nadam optimizer		Adam optimizer	
	Kappa value	MCC value	Kappa value	MCC value
EM1	0.9438	0.9446	0.9199	0.9184
EM2	0.9519	0.9523	0.9519	0.9523
EM3	0.9038	0.9030	0.9118	0.9125
EM4	0.9278	0.9295	0.8958	0.8966

Regardless of the optimizer used, EM2 performed exceptionally well, displaying high Kappa values that indicate a significant degree of agreement between its predicted classifications and the actual results (Nadam: κ=0.9519; Adam: κ=0.9519). These findings imply that when it came to correctly classifying observations, EM2 consistently performed better than the others. Even with slightly lower Kappa values (Adam: κ=0.9118; Nadam: κ=0.9038), EM1 showed a good degree of agreement.

Higher Kappa values are indicative of more robust and consistent predictive performance, offering practitioners and researchers insightful information about how well ensemble models perform under various optimization schemes.

The MCC results provide more information about the models’ performance. In addition to consistently displaying high Kappa values, EM2 also showed remarkable MCC values, highlighting its resilience in producing precise predictions (MCC = 0.9523 for Adam and MCC = 0.9523 for Nadam).

EM1 also displayed the second-highest performance in this criterion. The subtle variations in MCC values between the ensemble models and optimizers offer a thorough grasp of their predictive capacities. Models with higher MCC values are better able to predict outcomes accurately in a variety of classes. When paired with Kappa values, these MCC results help provide a more complete evaluation of the ensemble models.

#### Graphical evaluation.

The performance of the positive class is typically the minority or less frequent class and is the focus of precision-recall curves. More useful metrics for evaluating how well a model recognizes instances of the positive class are recall (a.k.a. true positive rate or sensitivity) and precision (a.k.a. positive predictive value). These metrics make it possible to track how the decision threshold is adjusted and how the trade-off between recall and precision changes.

Fig 17 provided an overview of the precision vs. recall performance evaluation of our ensemble models, whereas Figs 17a–17d showed the Adam optimizer curves and Figs 17e–17h the Nadam optimizer curves. The illustrated graphs also showed that EM2 with Adam

optimizer performed better than the other models. For every class, an additional examination of the precision-recall performance was conducted.

Impressively, the Aedes albopictus landing class achieved a 96% recall value and 100% precision. The Aedes aegypti landing class, on the other hand, showed a higher true positive rate than the positive predicted value, with a precision value of 92% and a recall value of 96%. With a sensitivity rate of 91%, EM2 positively predicted 95% of the Aedes aegypti smashed class. The precision and recall values of the Aedes albopictus smashed class were 93% and 100%, respectively. As seen in Fig 17b, the Culex quinquefasciatus landing class showed a balance between precision and recall values. For this class, EM2 achieved a 97% true positive rate and positive prediction rate. Finally, the Culex quinquefasciatus smashed class and attained an astounding 100% precision and 95% recall.

In summary, our findings suggested that EM2 equipped with the Adam optimizer is a dependable and efficient instrument for precisely forecasting mosquito species. EM2, with Nadam optimizer, performed the same, but for the Aedes albopictus smashed class, the precision value increased by 2%, reaching 93% from 91%.

Receiver operating characteristic (ROC) curves are generated to visually represent the relationship between sensitivity and specificity for different cut-off points in experimental combinations. From ROC curves, we also calculated the area under the ROC curve (AUC) values for all the classes. The maximum possible AUC value is 1.0. The higher the AUC value, the better the classifier.

The ROC curve analysis for the Adam and Nadam optimizer of the four ensemble models with six different mosquito classes is demonstrated in Fig 18. In Figs 18a–18d, the

performance of the Adam optimizer is shown, while in Figs 18e–18h, that of the Nadam optimizer is demonstrated.

A detailed examination of the AUC performance metric in the complex field of mosquito classification models reveals interesting trends among various ensembles and optimizers.

Aedes albopictus landing, where consistently high scores of 0.98 were achieved by EM1, EM2, and EM3 with the Adam optimizer. However, EM4, using the Nadam optimizer, experienced a considerable drop, recording a decreased landing score of 0.88.

Shifting our focus to the Aedes aegypti landing, the pinnacle was achieved at an outstanding 0.99 with EM1 using the Nadam optimizer. Yet, the journey for EM3 was challenging, as it struggled to gain only a 0.93 success rate, irrespective of whether it was the Adam or Nadam optimizer.

Aedes aegypti smashed, on the other hand, exhibited an impressive 0.95 score with EM2 in both Nadam and Adam optimizers. In contrast, EM1 lagged at 0.91, particularly in the Nadam optimizer.

The saga continues with Aedes albopictus smashed, showcasing remarkable performance at 0.99 with EM2 and EM4 in Adam, as well as EM1 and EM2 in Nadam optimizers. However, a drop in performance was marked with EM3 and EM4 in the Nadam optimizer, alongside EM1 in Adam, showing comparatively lower scores at 0.97.

Culex quinquefasciatus landing emerged as a strong performer, especially with EM2, earning a commendable 0.98 in both the Nadam and Adam optimizers. Yet, EM4 with the Adam optimizer experienced a fall, dropping to 0.94.

Finally, the class Culex quinquefasciatus smashed reached its peak at 0.98 with EM1, EM2, and EM4 in Adam, as well as EM2 and EM3 with the Nadam optimizer. However, EM1 with the Nadam optimizer demonstrated a relatively lower value of 0.96.

After a thorough examination of the mosquito classification models, EM2 has proven to perform exceptionally well in a variety of scenarios and classes. This ensemble model consistently attained outstanding peaks of 0.99 in Aedes aegypti landing, 0.95 in Aedes aegypti smashed, and 0.99 in Aedes albopictus smashed.

### Results on the second dataset (“Vector Mosquito”)

We mentioned earlier that for the purpose of classifying mosquito species, we employed four ensemble models. Notably, in our prior evaluation of the “Mosquito on Human Skin” dataset, Ensemble Model 2 (EM2) proved to be an outstanding performer. Encouraged by its promising performance, we also applied it to the “Vector Mosquito” dataset by Park *et al*. [7] (https://github.com/jypark1994/MosquitoDL). The dataset contains 3,600 images and 8 classes, but the authors grouped three mosquito species into a single non-vector class since they are considered as less potential vectors transmitting infectious diseases.

In this sub-section, we explore the experimental results of EM2 on Park *et al*.’s dataset (hereby named the “second dataset”) for vector Mosquito classification, aiming to understand how well it performs for this particular dataset. Following the general experimental setup of Park *et al*. [7], the dataset is split into 80% (2,880 images) for training and 20% (720 images) for validation (a.k.a. testing).

Table 10 represents the performance of vector mosquito classification of EM2 on this second dataset.

**Table 10:** Performance metrics for vector mosquito classification of on the second dataset.

Evaluation metric	Value
Precision	98%
Recall	98%
F1-score	98%
Accuracy	98.32%
Kappa	0.9798
MCC	0.9798

The performance metrics of EM2 on the second dataset demonstrated very good results across various evaluation parameters. Precision, recall, and F1-score were all significantly high at 98%, showing the model’s ability to classify vector mosquitoes accurately. The precision metric indicated the proportion of accurately predicted positive instances out of the total predicted positives, highlighting the model’s low false-positive rate. Similarly, the high recall underscored the model’s ability to capture a large portion of actual positives, strengthening its effectiveness in identifying vector mosquito classification. The F1-score, which balanced precision and recall, further emphasized the robust performance of EM2 on the Vector mosquito data.

The accuracy of 98.32% exhibited the overall correctness of the model’s predictions, showcasing its proficiency in classifying vector mosquitoes classification accurately. This increased accuracy rate is indicative of the model’s generalization ability on the Vector mosquito dataset, suggesting its adaptability to various instances within the data.

Kappa and MCC both stand at an exceptional 0.9798. These metrics evaluated the agreement between predicted and actual classifications, with higher values reflecting substantial concordance. EM2’s Kappa and MCC values indicated its reliability in consistently making accurate predictions on the Vector mosquito classification.

In our analysis of vector mosquito data, EM2 performed outstandingly across various classes in terms of AUC value, which is demonstrated in Fig 20. ROC curves are generally operated to visually represent the relationship between sensitivity and particularity for different cut-off points in experimental combinations. We also calculated the AUC value for all classes. The scores provided a sophisticated insight into its classification ability. The model performed for Aedes albopictus (0.9900), Aedes vexans (0.9757), Anopheles sinensis (0.9941), Culex pipiens (0.9943), and Culex tritaeniorhynchus (0.9870). Notably, the model performed at a perfect value of 1.0000 for non-vectors.

EM2 demonstrated robust learning capabilities, as illustrated by its superior performance when classifying vector mosquitoes.

After plotting the ROC curves and calculating the AUC values for particular mosquito classes, we proceeded to illustrate the precision and recall curves for different mosquito classes, including Aedes albopictus, Aedes vexans, Anopheles sinensis, Culex pipiens, Culex tritaeniorhynchus, and Non-vectors which have been represented in Fig 21. This visualization presented a brief overview of the classification model’s performance, showing the trade-off between precision and recall, also important metrics for evaluating the effectiveness of classification models and play a vital role in acquiring accurate identification of a specific class (recall) and minimizing the occurrence of false positives (precision).

Aedes albopictus showed an incredible precision of 1.00, indicating that this species is nearly always correct when predicted. However, the recall of 0.98 indicated that the model may miss a small proportion of instances.

Aedes vexans, with a precision of 0.98 and a recall of 0.95, demonstrated a high precision but comparatively lower recall. It may be suggested that while the model is adept at perfectly classifying Aedes vexans, it tends to miss some occurrences, potentially due to overlapping features with other classes or inherent class ambiguity.

Anopheles sinensis showed excellent precision and recall values of 0.98 and 0.99, respectively, demonstrating high accuracy in both accurately identifying instances and minimizing false positives. This suggested a strong performance in distinguishing Anopheles sinensis from other classes.

Culex pipiens exhibited a slightly lower precision of 0.94, meaning a small fraction of false positives. However, it gained a perfect recall of 1.00, showing that the model effectively captures all instances of Culex pipiens.

Culex tritaeniorhynchus displayed an outstanding precision of 0.99 and a recall of 0.98, showcasing a high degree of accuracy in classification. The lower recall may be due to specific instances that share features with other mosquito classes.

Non-vectors demonstrated perfect precision and recall, both at 1.00, showing flawless performance in correctly identifying instances and avoiding false positives. This is expected, as non-vectors are more distinguishable from the other mosquito classes.

Variations in accuracy and recall performance across mosquito classes may result from species-specific differences, overlapping traits, or the inherent challenges of class ambiguity. Model accuracy and the ability to balance recall are important for practical applications, and understanding the factors contributing to these variations is essential to refining the model and improving overall performance.

The confusion matrix is shown in Fig 22. Here, the model error rate and misclassified samples are visualized. From the figure, Aedes albopictus was mainly classified correctly with 98 predictions, but misclassifications occurred 2% with 1 different from Anopheles sinensis, and 1 was considered as Culex pipiens. On the other hand, Aedes vexans exhibited more misclassification (4.51%), where 1 individual was misclassified as Anopheles sinensis, 4 as Culex pipiens, and 1 as non-vectors. Anopheles sinensis recorded a higher score in classification, having 118 correct and only 1 misclassification as Culex pipiens (0.84 percent error). Culex pipiens and non-vectors were perfectly classified (0% misclassification), while Culex tritaeniorhynchus had 120 true classifications but was misclassified 2 times as Aedes vexans and 1 time as Culex pipiens, hence having a 2.43% misclassification rate. The results suggest that despite the fact that the model did very well in general, Aedes vexans had the highest misclassification rate, with the misclassification primarily against Culex pipiens.

## Discussions

In our study, we first classify the mosquito species using a transfer learning model. The transfer learning models’ performance was promising, but when we started to analyze deeply, we planned to apply the ensemble model. So, we did not combine any specific models to make the ensemble model. When making the ensemble model, we considered all possible combinations. We found that after applying the first ensemble model, the performance is significantly increased. In our pre-trained model, the best-performing model was DenseNet-121. The study showed that the ensemble model performed well when we did not take the DenseNet-121 model.

Our proposed Ensemble Model 2 (EM2) performed best, as we mentioned earlier. The model outperformed all models from all criteria. Ensemble models perform better on classification tasks, especially when they combine pre-trained models. The ensemble attains improved accuracy and broader dataset generalization by utilizing the complementary features of multiple models. One common issue in the DL is overfitting, which is lessened by the ensemble approach. By allowing each model in the ensemble to capture a distinct aspect of the data, the likelihood of overly relying on the peculiarities of the training set is decreased.

DL architecture-based models, for example, are pre-trained and can learn complex feature representations from large, complicated datasets. Combining different representations helped the ensemble identify subtle patterns that are important for differentiating between mosquito species. Pre-trained models capture generic features that are helpful for a variety of tasks because they are trained on large and diverse datasets. By utilizing transfer learning, our model potentially improved performance on mosquito species classification by utilizing knowledge acquired in unrelated domains. However, Ensemble models can be computationally demanding during both training and inference, particularly if they include pre-trained DL models. For environments with limited resources or real-time applications, this could present difficulties. As we were using high-level GPU, we balanced the complexity of our models with the available resources. Acknowledging limitations associated with smashed specimens, we worked greatly on these samples. Importantly, the implications of our findings extend to disease surveillance, emphasizing the relevance of accurate species identification even when dealing with compromised specimen conditions. Apart from examining Aedes aegypti, Aedes albopictus, and Culex quinquefasciatus in their smashed state, we also expanded. We also worked on another dataset that included vector mosquitoes. This dataset offered a chance to investigate how well our classification techniques applied and generalized to a wide variety of vector species.

EM2 performed well in our research with Adam optimizer, and we applied this approach to this dataset. Our study intended to offer a comprehensive understanding of the ensemble model’s capabilities and limitations in the context of broader mosquito vector classification by addressing a wider range of vector species. We have compared our model with previous state-of-the-art models that were applied to classify mosquito species and vector mosquitoes.

### Strengths and limitations

Here, we will discuss the strengths and limitations of our proposed method.

#### Strengths.

The two main strengths of our proposed method are as follows.

**Reducing complexity:** Although ensemble-based models are recognized for their increased accuracy, real-time applicability is questioned due to their computational cost. Even for delicate activities like vector mosquitoes, we prioritize gaining better classification performance, and we are open to investigating efficient learning strategies to lower computational costs. To achieve our desired results, the ensemble model that we have suggested makes use of multiple customized Deep Convolutional Neural Networks (DCNNs) that are adapted to different learning constraints. With 138 million parameters, the original pre-trained VGG-16 model is known for its complicated architectural design; however, this complexity has been significantly reduced by our fine-tuning procedure. This decrease in the number of parameters is a common occurrence seen in all of the pre-trained models used as base learners in our study.

For a comprehensive analysis of the parameters for each base model, we have presented Table 4. A typical deep ensemble model usually entails using many CNN designs and averaging the predictions that result from them. Our work seeks to set itself apart, nevertheless, by proving the effectiveness of ensembles based on transfer learning while also cutting down on computing complexity. We reduced the requirement for extensive training from the start by leveraging the knowledge gained from pre-trained models by utilizing the power of transfer learning.

We have found that our EM2 model (InceptionV3 + ResNet-50 + VGG-16) performed the best. The large number of trainable parameters for DenseNet-121 (over 3 million, ref. Table 4) is not in this best-performing combination. So, our model not only improves performance but also significantly lowers its computing overhead after customizing the pre-trained models.

**Prediction quality and robustness:** The proposed method did not suffer from any “accuracy fallacy” or “accuracy paradox.” The term accuracy fallacy/paradox refers to a situation in which a model achieves a high accuracy but is unable to predict correctly. This phenomenon occurs when a model performs well overall but finds it difficult to classify instances correctly, especially in datasets that are imbalanced or when misclassification costs differ between classes. The accuracy paradox draws attention to the drawbacks of using accuracy as the only performance metric. This is because accuracy may not accurately represent the efficacy of the model, particularly in practical applications where precise predictions are crucial.

To acquire a more thorough grasp of the model’s overall prediction quality and robustness, it is necessary to take into account alternative metrics such as AUC, F1-score, precision, recall, MCC, and Kappa. The proposed model has also been evaluated on those measurement criteria, and the analysis of the outcomes shows that the model can overcome the problem of accuracy fallacy. As stated before, the dataset is imbalanced, but our model does not exhibit much bias against minority classes. The confusion matrices, precision-recall curves, and ROC curves showed excellent class-wise performances of the proposed method graphically.

#### Limitations.

On the other hand, our proposed method has its limitations.

**Dependence on transfer learning:** Because our model depends on pre-trained networks, its initial knowledge may derive from datasets that are not entirely relevant to our particular topic. By utilizing past information, transfer learning dramatically improves performance, yet it also restricts the model’s capacity to learn features that are unique to a given domain. Predictive performance may suffer when our dataset differs significantly from the ones used to pre-train the models, requiring further fine-tuning or even starting over.

**Data imbalance challenges:** Although our model has demonstrated encouraging outcomes in managing data imbalance, it is not completely impervious to the difficulties presented by severely skewed datasets. The suggested method’s performance may be further improved if the data distribution is more evenly distributed.

**Interpretability issues:** Interpretability/explainability is still a major challenge even though our model performs exceptionally well in terms of accuracy and other performance measures. Ensemble models typically operate as “black boxes,” making it challenging to comprehend the decision-making process, particularly for those based on deep learning architectures. This lack of transparency may be an issue when implementing models in industries where interpretability is critical, such as healthcare, where knowing the reasoning behind predictions is just as vital as the predictions themselves.

### Comparison with other state-of-the-art methods

Ong and Ahmed [16] compiled the first dataset used in our research. They used web-based DCNN (namely, Google Teachable Machine 2.0 [29]) in their research and achieved 92.56% accuracy. However, our EM2 model indicatively outperformed their model, obtaining 96% validation accuracy for classifying the mosquito species by using the ensemble learning strategy.

Kumar *et al*. [17] used the same dataset created by Ong and Ahmed [16] and applied 6 different DL algorithms, namely, simple DCNN, EfficientNetB7, MobileNetV2, DenseNet121, XceptionNet, and ResNet152V2. They observed that the simple DCNN with hyperparameter tuning provides the best accuracy of 91%. Again, our proposed model, EM2, indicatively achieves a higher accuracy.

For vector mosquito classification, Park *et al*. [7] employed transfer learning VGG-16 mode. In the dataset they collected, there were 3,600 samples. The authors achieved 97.19% accuracy. They evaluated the model using just the accuracy measure, although precision, recall, and F1-score are important evaluation metrics for an imbalanced dataset. Their result showed that the model confused a maximum of 6.26% times to differentiate between classes. Our analysis showed in Fig 22 that the proposed EM2 exhibits less confusion among the different classes. In particular, there is no confusion at all for the non-vector class. We achieved 98.32% accuracy on this dataset with our proposed model.

Table 11 gives indicative comparisons of our proposed model (EM2) with the other state-of-the-art methods on the two datasets. The comparisons are also displayed graphically in Figs 23 and 24.

**Fig 19:**
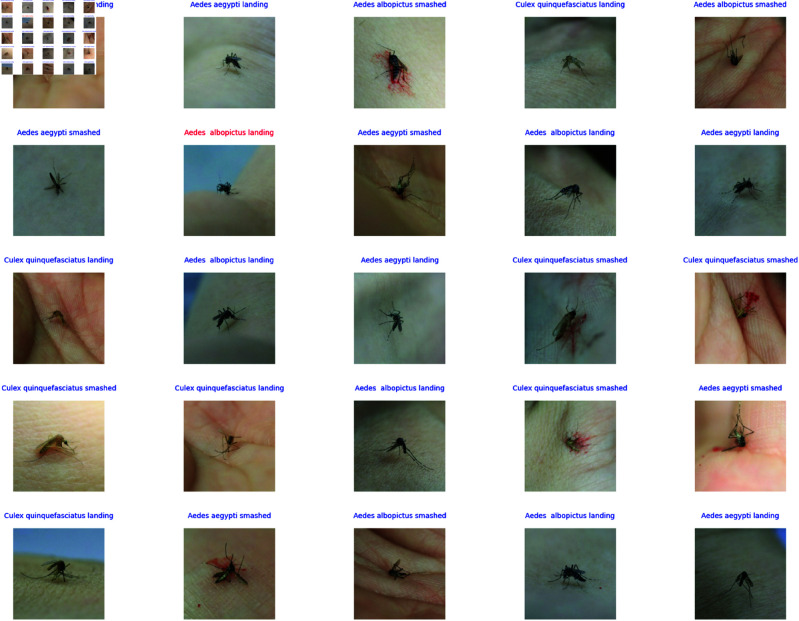
Sample outputs of EM2. The blue text color indicates that the model could accurately predict the class, whereas the red text color means a wrong prediction. (The images are reproduced with permission by the original images’ owners [16]).

**Fig 20:**
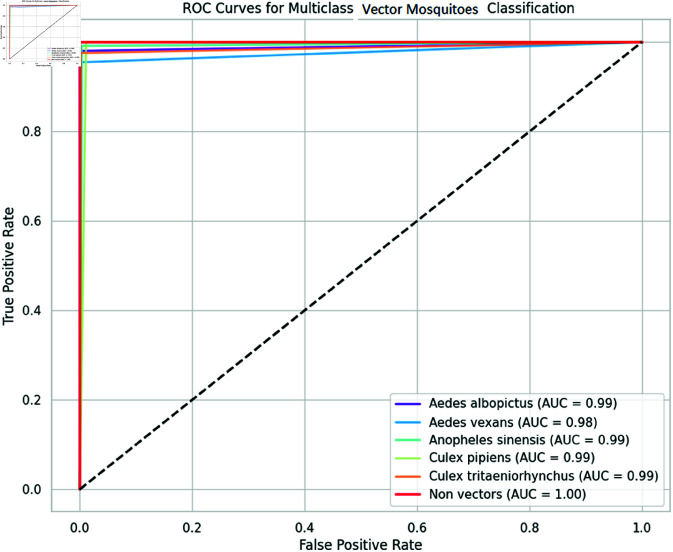
ROC curves for vector mosquito classification on the second dataset.

**Fig 21:**
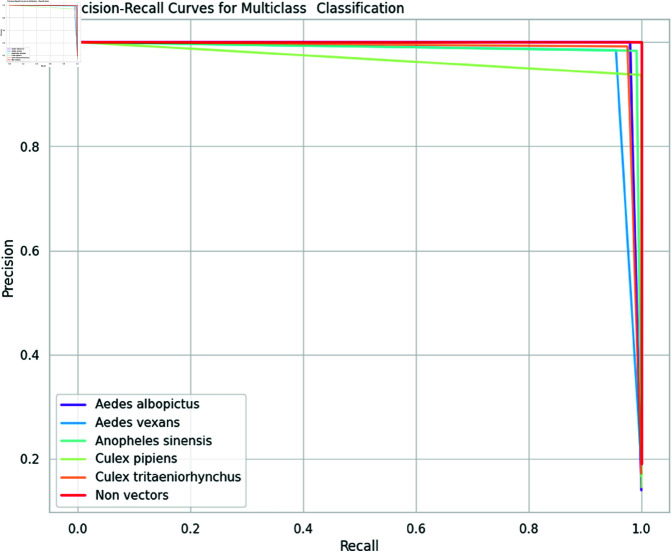
Precision-recall curves for vector mosquito classification on the second dataset.

**Fig 22:**
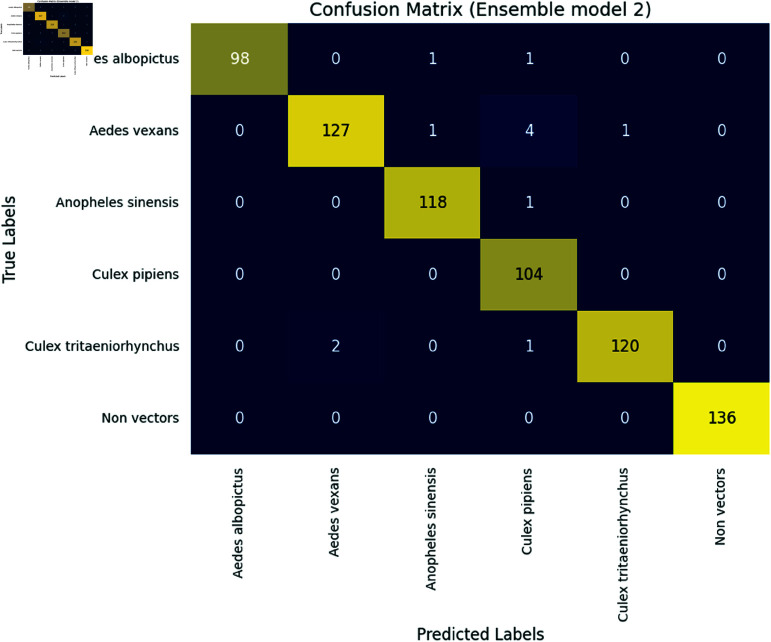
Confusion matrix for vector mosquito classification on the second dataset.

**Fig 23:**
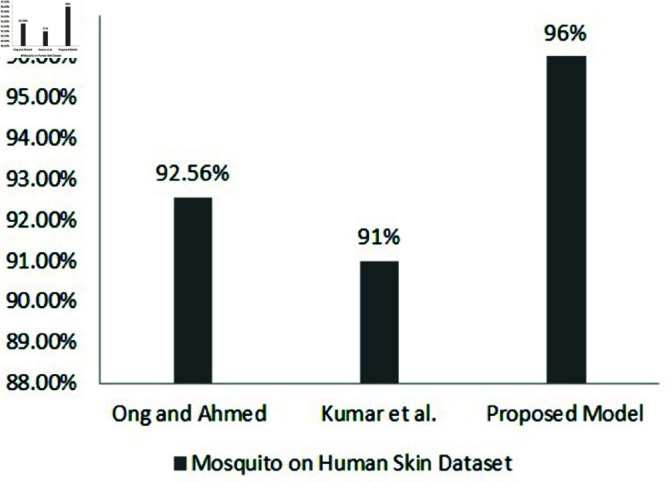
Indicative performance comparison on “Mosquito on Human Skin” dataset.

**Fig 24:**
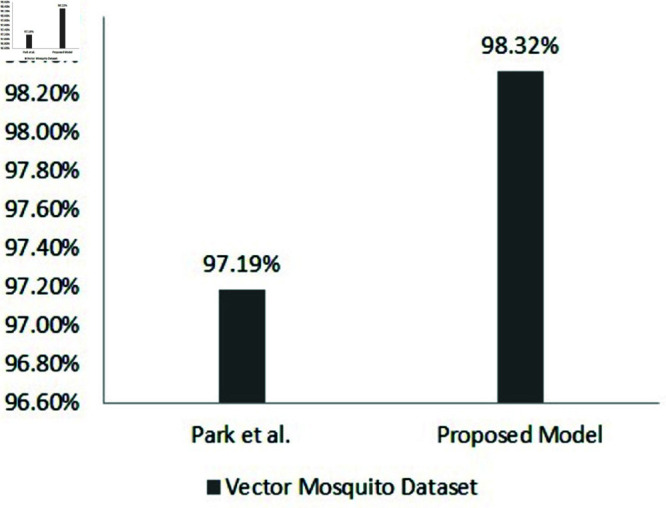
Indicative performance comparison on “Vector Mosquito” dataset.

**Table 11:** Comparisons of the accuracy results of our proposed model (EM2) with those of the other state-of-the-art methods (as reported in their respective publications). (Caveat: here, the comparisons are merely indicative because different experimental protocols were used.)

Method	Train:validation split	Accuracy	Remark
Mosquito on Human Skin dataset [16]			
Ong and Ahmed [16]	2100:900 (70:30)	92.56%	Preprocessed data was used.
Kumar *et al*. [17]	2100:900 (70:30)	91%	Preprocessed data was used.
Proposed model (EM2)	1350:150 (90:10)	96%	Raw data was used.
Vector Mosquito dataset [7]			
Park *et al*. [7]	2880:720 (80:20)	97.19%	5-fold cross-validation was used.
Proposed model (EM2)	2880:720 (80:20)	98.32%	Single split was used.

As the proposed model is benchmarked against previous research works that used the same datasets, it is observed that we achieved superior performances compared to them (as summarized in Figs 23 and 24).

Our EM2 model is well-generalized with neither overfitting nor underfitting observed, whereas many of the previous models faced overfitting or underfitting issues [15,20]. Furthermore, the proposed model reduces the computational complexity, although we are using an ensemble strategy. We have found some of the models applied in previous research showed some biased results for minority classes, whereas the proposed model is not biased as in [6].

In Goodwin *et al*. [6], the dataset contains 67 species of mosquitoes in total. However, they chose 20 species to be deemed known for their study based on a number of factors, including the number of specimens in the database, the species’ medical significance, and the distribution of known and unknown species in the class. Sixteen known species were present in each of the five data folds that were used to train the model; the remaining species were regarded as unknown for each fold. As a result, they took into account 20 classes and the unbalanced conditions in the photos for their classification task. Ae. albopictus contains 692 samples, compared to 343 for Cx. erraticus. Their research indicates that they have 71% accuracy for Cx. erraticus and 99% accuracy for this class.

Additionally, many of the prior research works used datasets with a limited number of classes (such as 2 or 3) [13-15,25]. Moreover, since their datasets contained only a very limited number of samples, some of the previous works had to rely on data augmentation techniques to increase the sample size [25]. On the other hand, in our research, we used two datasets with 6 classes in each of them with a sizeable number of samples. So, our proposed methodology is advanced in several factors, such as data quantity, data diversity, robustness, performance, and reducing computational complexity.

## Conclusion

In the present work, we proposed a novel methodology, TransembleNet, for classifying mosquito species. We used a combination of pre-trained CNN models with two distinct optimizers and incorporated data augmentation techniques to enhance comprehensive generalization. Our emphasis on vector mosquitoes, especially in the context of diseases such as malaria, dengue, and Zika, introduces a significant practical relevance to our work. The success of our approach lies in exhibiting the efficacy of deep convolutional neural networks for accurate classification of mosquito species, mainly when confronted with the challenges of high inter-species similarity and intra-species variations.

On the “Mosquito on Human Skin” dataset, the proposed Ensemble Model 2 (EM2) appeared as the top performer, acquiring an impressive 96% precision, recall, F1-score, and accuracy, outperforming previous research benchmarks. On another dataset, namely, “Vector Mosquito”, with a very high 98.32% accuracy, EM2 also outperformed the previous research works.

### Future works

In this research, we worked on 3 species of mosquitoes and their landing/smashed conditions. Gathering a dataset that includes more species is a promising research direction. Expanding the dataset could help in assessing the robustness of the model in more diverse real-world scenarios. The dataset could also be organized based on the different regions. Malaria and dengue are life-threatening for third-world countries. Thus, more extensive research is needed for the responsible species to control and understand the disease patterns.

The proposed TransembleNet scheme, while specifically optimized for vector mosquito classification, holds broader potential for various classification tasks across different domains. When paired with transfer learning from pre-trained models, its ensemble-based methodology offers a versatile framework that can be used for various datasets where high accuracy and robustness are necessary. TransembleNet, for instance, might be used in environmental monitoring to find patterns in satellite or aerial images, or it could be used in medical image analysis to discover minute alterations in features.

Furthermore, its incorporation into the current public health surveillance systems might greatly improve the efficiency of tracking and responding to diseases. Especially in isolated or resource-constrained places, authorities might monitor vector-borne diseases in real time by integrating TransembleNet into mobile health applications or edge devices. The model is a dynamic tool for changing settings because of its modular nature, which enables it to learn from fresh data continuously.

In order to address the issue of interpretability/explainability, we have a plan to explore Explainable Artificial Intelligence (XAI) techniques for images [54] and select the suitable ones for our task. In the future, TransembleNet’s resilience and adaptability, when incorporated with its interpretability, will have a great potential to impact applications beyond vector mosquito classification, providing promising capabilities for a variety of classification challenges and public health applications.

Lastly, the introduction of TransembleNet into present public health surveillance systems can magnify the capacity for tracking diseases and responding to them. Specifically, integration with mobile health applications and edge computing [55] devices would enable real-time surveillance of vector-borne diseases in remote or resource-poor areas. By deploying the model on edge devices such as smartphones, drones, or IoT-enabled traps, mosquito classification can be performed locally without relying on constant cloud connectivity. This would allow rapid on-site analysis, reducing latency and enabling immediate decision-making for vector control strategies. Additionally, edge computing can enhance privacy by processing sensitive health data locally, reducing the risk of data breaches while ensuring continuous monitoring and disease prevention.

## Code availability

All the models applied in the research can be found in the publicly accessible GitHub repository: https://github.com/abdullahamruf/Mosquito. All are open source and publicly available.

## Dedication

This research paper is dedicated to the loving memory of Mr. **Md. Emadul Haque**, the beloved father of Md. Mahmudul Haque, who passed away in 2023 after battling dengue. His wisdom, kindness, and unwavering support continue to inspire us. May his soul rest in eternal peace.

## References

[1] World Health Organization. Vector-borne diseases; 2020. Available from: https://www.who.int/news-room/fact-sheets/detail/vector-borne-diseases

[2] Morchón R, Bueno-Marí R, Bravo-Barriga D. Biology, control and zoonotic role of disease vectors. Pathogens. 2023;12(6):797. doi: 10.3390/pathogens12060797 37375487 PMC10304399

[3] Ogunlade S. T. Modeling the potential of introducing different Wolbachia-infected mosquitoes to control Aedes-borne arboviral infections. James Cook University; 2023.

[4] Anoopkumar AN, Aneesh EM. A critical assessment of mosquito control and the influence of climate change on mosquito-borne disease epidemics. Environ Dev Sustain. 2022;24(6):8900–29.

[5] Zhao D-Z, Wang X-K, Zhao T, Li H, Xing D, Gao H-T, et al. A Swin transformer-based model for mosquito species identification. Sci Rep. 2022;12(1):18664. doi: 10.1038/s41598-022-21017-6 36333318 PMC9636261

[6] Goodwin A, Padmanabhan S, Hira S, Glancey M, Slinowsky M, Immidisetti R, et al. Mosquito species identification using convolutional neural networks with a multitiered ensemble model for novel species detection. Sci Rep. 2021;11(1):13656. doi: 10.1038/s41598-021-92891-9 34211009 PMC8249627

[7] Park J, Kim DI, Choi B, Kang W, Kwon HW. Classification and morphological analysis of vector mosquitoes using deep convolutional neural networks. Sci Rep. 2020;10(1):1012. doi: 10.1038/s41598-020-57875-1 31974419 PMC6978392

[8] Favret C, Sieracki JM. Machine vision automated species identification scaled towards production levels. Syst Entomol. 2016;41(1):133–143. doi: 10.1111/syen.12146

[9] Yoo H-J. Deep convolution neural networks in computer vision: A review. IEIE Trans Smart Process Comput. 2015;4(1):35–43. doi: 10.5573/ieiespc.2015.4.1.035

[10] Cecotti H. Rotation invariant descriptors for galaxy morphological classification. Int J Mach Learn Cybernet. 2020;11(8):1839–1853. doi: 10.1007/s13042-020-01075-w

[11] Wu X, Zhang YT, Lai KW, Yang MZ, Yang GL, Wang HH. A novel centralized federated deep fuzzy neural network with multi-objectives neural architecture search for epistatic detection. IEEE Trans Fuzzy Syst. 2024.

[12] Hall M, Tamïr D. Mosquitopia: The place of pests in a healthy world. Taylor & Francis; 2022. 36260711

[13] Adhane G, Dehshibi MM, Masip D. A deep convolutional neural network for classification of Aedes albopictus mosquitoes. IEEE Access. 2021;9:72681–72690. doi: 10.1109/access.2021.3079700

[14] Ong S-Q, Ahmad H, Nair G, Isawasan P, Majid AHA. Implementation of a deep learning model for automated classification of Aedes aegypti (Linnaeus) and Aedes albopictus (Skuse) in real time. Sci Rep. 2021;11(1):9908. doi: 10.1038/s41598-021-89365-3 33972645 PMC8110999

[15] Jeyakodi G, Agarwal T, Bala PS. mAedesID: Android application for Aedes mosquito species identification using convolutional neural network. arXiv preprint arXiv:230507664. 2023.

[16] Ong S-Q, Ahmad H. An annotated image dataset for training mosquito species recognition system on human skin. Sci Data. 2022;9(1):413. doi: 10.1038/s41597-022-01541-w 35840589 PMC9287291

[17] Kumar CSA, Maharana AD, Krishnan SM, Hanuma SSS, Sowmya V, Ravi V. Mosquito on human skin classification using deep learning. In: Innovations in machine and deep learning: Case studies and applications. Springer; 2023. p. 193–212.

[18] Hosna A, Merry E, Gyalmo J, Alom Z, Aung Z, Azim MA. Transfer learning: A friendly introduction. J Big Data. 2022;9(1):102. doi: 10.1186/s40537-022-00652-w 36313477 PMC9589764

[19] Wu X, Wang H, Tan W, Wei D, Shi M. Dynamic allocation strategy of VM resources with fuzzy transfer learning method. Peer-to-Peer Netw Appl. 2020;13(6):2201–2213. doi: 10.1007/s12083-020-00885-7

[20] Azman MIABZ, Sarlan AB. Aedes larvae classification and detection (ALCD) system by using deep learning. In: Proceedings of the 2020 international conference on computational intelligence (ICCI). 2020:179–184. doi: 10.1109/icci51257.2020.9247647

[21] Arista-Jalife A, Nakano M, Garcia-Nonoal Z, Robles-Camarillo D, Perez-Meana H, Arista-Viveros HA. Aedes mosquito detection in its larval stage using deep neural networks. Knowl-Based Syst. 2020;189:104841. doi: 10.1016/j.knosys.2019.07.012

[22] De Silva W, Jayalal S. Dengue mosquito larvae identification using digital images. In: Proceedings of the 2020 international research conference on smart computing and systems engineering (SCSE). IEEE; 2020. p. 31–6.

[23] Garcia PSC, Martins R, Coelho GLLM, Cámara-Chávez G. Acquisition of digital images and identification of Aedes aegypti mosquito eggs using classification and deep learning. In: Proceedings of the 2019 32nd SIBGRAPI conference on graphics, patterns and images (SIBGRAPI). IEEE; 2019. p. 47–53.

[24] Neto AA, Bezerra FN, Sousa CEB, Neto MAV, e Silva JHA, de Azevedo DAA. Identification of the Aedes aegypti/albopictus mosquito using digital image processing techniques. In: Proceedings of the 2020 IEEE 5th international conference on signal and image processing (ICSIP). IEEE; 2020. p. 518–23.

[25] Akter M, Hossain MS, Ahmed TU, Andersson K. Mosquito classification using convolutional neural network with data augmentation. In: International conference on intelligent computing & optimization. Springer; 2020. p. 865–79.

[26] Rustam F, Reshi AA, Aljedaani W, Alhossan A, Ishaq A, Shafi S, et al. Vector mosquito image classification using novel RIFS feature selection and machine learning models for disease epidemiology. Saudi J Biol Sci. 2022;29(1):583–594. doi: 10.1016/j.sjbs.2021.09.021 35002454 PMC8717167

[27] De Los Reyes AMM, Reyes ACA, Torres JL, Padilla DA, Villaverde J. Detection of Aedes Aegypti mosquito by digital image processing techniques and support vector machine. In: Proceedings of the 2016 IEEE region 10 conference (TENCON). 2016. p. 2342–5. doi: 10.1109/tencon.2016.7848448

[28] Motta D, Santos AÁB, Winkler I, Machado BAS, Pereira DADI, Cavalcanti AM, et al. Application of convolutional neural networks for classification of adult mosquitoes in the field. PLoS ONE. 2019;14(1):e0210829. doi: 10.1371/journal.pone.0210829 30640961 PMC6331110

[29] Carney M, Webster B, Alvarado I, Phillips K, Howell N, Griffith J, et al. Teachable machine: Approachable web-based tool for exploring machine learning classification. In: Extended abstracts of the 2020 CHI conference on human factors in computing systems; 2020. p. 1–8. doi: 10.1145/3334480.3382839

[30] Krizhevsky A, Hinton G, et al. Learning multiple layers of features from tiny images. Canada: University of Toronto; 2009.

[31] Deng J, Dong W, Socher R, Li LJ, Li K, Fei-Fei L. Imagenet: A large-scale hierarchical image database. In: Proceedings of the 2009 IEEE conference on computer vision and pattern recognition (CVPR). IEEE; 2009. p. 248–55.

[32] Qian Y. Performance comparison among VGG16, InceptionV3, and resnet on galaxy morphology classification. J Phys: Conf Ser. 2023;2580(1):012009. doi: 10.1088/1742-6596/2580/1/012009

[33] Szegedy C, Liu W, Jia Y, Sermanet P, Reed S, Anguelov D, et al. Going deeper with convolutions. In: Proceedings of the IEEE conference on computer vision and pattern recognition (CVPR); 2015. p. 1–9.

[34] Koonce B, Koonce B. ResNet 50. In: Convolutional neural networks with Swift for Tensorflow: Image recognition and dataset categorization. Springer; 2021. p. 63–72.

[35] He K, Zhang X, Ren S, Sun J. Deep residual learning for image recognition. In: Proceedings of the IEEE conference on computer vision and pattern recognition (CVPR); 2016. p. 770–8.

[36] Zhu Y, Newsam S. Densenet for dense flow. In: Proceedings of the 2017 IEEE international conference on image processing (ICIP). IEEE; 2017. p. 790–4.

[37] Huang G, Liu Z, Van Der Maaten L, Weinberger KQ. Densely connected convolutional networks. In: Proceedings of the IEEE conference on computer vision and pattern recognition (CVPR); 2017. p. 4700–8.

[38] Tammina S. Transfer learning using VGG-16 with deep convolutional neural network for classifying images. Int J Sci Res Publ. 2019;9(10):143–50. doi: 10.29322/ijsrp.9.10.2019.p9420

[39] Simonyan K, Zisserman A. Very deep convolutional networks for large-scale image recognition. arXiv preprint arXiv:14091556. 2014.

[40] Maurício J, Domingues I, Bernardino J. Comparing vision transformers and convolutional neural networks for image classification: A literature review. Appl Sci. 2023;13(9):5521.

[41] Alzubaidi L, Zhang J, Humaidi AJ, Al-Dujaili A, Duan Y, Al-Shamma O, et al. Review of deep learning: concepts, CNN architectures, challenges, applications, future directions. J Big Data. 2021;8:1–74. doi: 10.1186/s40537-021-00444-8 33816053 PMC8010506

[42] Vallabhajosyula S, Sistla V, Kolli VKK. Transfer learning-based deep ensemble neural network for plant leaf disease detection. J Plant Dis Prot. 2022;129(3):545–58. doi: 10.1007/s41348-021-00465-8

[43] Mohammed A, Kora R. A comprehensive review on ensemble deep learning: Opportunities and challenges. J King Saud Univ Comput Inform Sci. 2023;35(2):757–74. doi: 10.1016/j.jksuci.2023.01.014

[44] Saleh H, Mostafa S, Alharbi A, El-Sappagh S, Alkhalifah T. Heterogeneous ensemble deep learning model for enhanced Arabic sentiment analysis. Sensors. 2022;22(10):3707. doi: 10.3390/s22103707 35632116 PMC9147256

[45] Chennamsetty S, Safwan M, Alex V. Classification of breast cancer histology image using ensemble of pre-trained neural networks. In: Image analysis and recognition: 15th international conference (ICIAR). Springer; 2018. p. 804–11.

[46] Nimmi K, Janet B, Selvan AK, Sivakumaran N. Pre-trained ensemble model for identification of emotion during COVID-19 based on emergency response support system dataset. Appl Soft Comput. 2022;122:108842. 35465357 10.1016/j.asoc.2022.108842 PMC9014641

[47] Maruf A, Fahim SH, Bashar R, Rumy RA, Chowdhury SI, Aung Z. Classification of freshwater fish diseases in Bangladesh using a novel ensemble deep learning model: Enhancing accuracy and interpretability. IEEE Access. 2024;12:96411–35. doi: 10.1109/access.2024.3426041

[48] S K, Inbarani HH. Ensemble pre-trained deep convolutional neural network model for classifying medical image datasets. In: Proceedings of the 2022 international conference on augmented intelligence and sustainable systems (ICAISS). 2022:121–8. doi: 10.1109/icaiss55157.2022.10011089

[49] Agarwal V. Destroy image classification by ensemble of pre-trained models; 2020. Available from: https://towardsdatascience.com/destroy-image-classification-by-ensemble-of-pre-trained-models-f287513b76

[50] Ju C, Bibaut A, van der Laan M. The relative performance of ensemble methods with deep convolutional neural networks for image classification. J Appl Stat. 2018;45(15):2800–18. doi: 10.1080/02664763.2018.1441383 31631918 PMC6800663

[51] Kingma DP, Ba J. Adam: A method for stochastic optimization. arXiv preprint arXiv:14126980. 2014.

[52] Dozat T. Incorporating Nesterov momentum into Adam. In: Proceedings of the 2016 international conference on learning representations (ICLR) - Workshop track; 2016. p. 1–4.

[53] Hassan E, Shams MY, Hikal NA, Elmougy S. The effect of choosing optimizer algorithms to improve computer vision tasks: A comparative study. Multimed Tools Appl. 2023;82(11):16591–633. doi: 10.1007/s11042-022-13820-0 36185324 PMC9514986

[54] van der Velden BHM, Kuijf HJ, Gilhuijs KGA, Viergever MA. Explainable artificial intelligence (XAI) in deep learning-based medical image analysis. Med Image Anal. 2022;79:102470. doi: 10.1016/j.media.2022.102470 35576821

[55] Rai N, Zhang Y, Villamil M, Howatt K, Ostlie M, Sun X. Agricultural weed identification in images and videos by integrating optimized deep learning architecture on an edge computing technology. Comput Electron Agric. 2024;216:108442. doi: 10.1016/j.compag.2023.108442

